# Proteostasis Rebalancing by LET‐607 Deficiency Promotes Longevity

**DOI:** 10.1111/acel.70620

**Published:** 2026-07-09

**Authors:** Haixiang Tong, Wei Li, Pangui Yuan, Feng Li, Qin Liu, Shanshan Pang, Haiqing Tang

**Affiliations:** ^1^ School of Life Sciences Chongqing University Chongqing China

**Keywords:** HSF‐1, longevity, proteostasis, unfolded protein response

## Abstract

Disruption of proteostasis is a hallmark of aging. Given that cellular resources are limited, this necessitates a coordinated orchestration of different proteostatic subsystems. Yet, the principles governing this process, including the potential role of trade‐offs, are not well defined. Here, we report a trade‐off between the endoplasmic reticulum unfolded protein response (UPR^ER^) and the cytosolic UPR (UPR^cyto^) in *C. elegans* that influences lifespan. We find that wild‐type animals maintain high UPR^ER^ activity but low UPR^cyto^ activity, a balance actively enforced by the transcription factor LET‐607 (ortholog of mammalian CREBH). Consequently, LET‐607 deficiency releases this trade‐off, causing a seesaw‐like rebalancing: UPR^ER^ activity decreases while UPR^cyto^ increases. Strikingly, this rebalancing contributes to longevity: animals lacking LET‐607 exhibited extended lifespan in a UPR^cyto^ dependent manner. Mechanistically, LET‐607 deficiency downregulates one‐carbon cycle, which provides the methyl donor S‐adenosylmethionine. This subsequently alleviates H3K9me‐mediated repression at the promoters of UPR^cyto^ genes, a process involving the regulators and readers of this histone mark, leading to UPR^cyto^ activation. Our study reveals a transcriptional mechanism that enforces a proteostatic trade‐off and demonstrates that evolutionarily acquired UPR balance in wild‐type animals is suboptimal for longevity, supporting the antagonistic pleiotropic theory of aging.

## Graphical abstract

LET‐607 enforces a trade‐off between UPR^ER^ and UPR^cyto^ in *C. elegans* , maintaining a proteostatic balance optimized for development but suboptimal for longevity. LET‐607 deficiency suppresses UPR^ER^ but activates UPR^cyto^ via attenuation of one‐carbon metabolism and H3K9 methylation, thereby enhancing cytosolic proteostasis and extending lifespan.

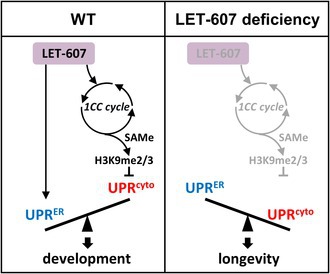

## 1 Introduction

The accumulation of misfolded proteins is a key driver of aging, and declining proteostasis is now recognized as a central hallmark of aging (López‐Otín et al. 2023). Strategies that enhance proteostasis have been consistently shown to extend lifespan in model organisms (Hipp et al. 2019; Klaips et al. 2018). Protein misfolding stress can occur in various cellular compartments, most notably the ER, cytoplasm, and mitochondria. To cope with this, cells have evolved compartment‐specific protein quality control systems known as the UPR, which operate in these three compartments to facilitate proper protein folding (Hetz et al. 2020; Shpilka and Haynes 2018; Acosta‐Alvear et al. 2025). Notably, activation of the UPR in any single compartment is sufficient to promote longevity in model organisms (Taylor and Dillin 2013; Imanikia et al. 2019; Durieux et al. 2011; Hsu et al. 2003; De‐Souza et al. 2023; Li et al. 2024), making the regulation of UPR a major focus in aging research.

However, as an integrated system, cells must not only regulate each UPR pathway independently but also orchestrate their activities collectively. Previous studies have elegantly demonstrated that perturbation of one UPR pathway can lead to the compensatory activation of another (Ruan et al. 2017; Wang and Chen 2015; Kim et al. 2016; Li et al. 2025). These responses are homeostatic mechanisms that are triggered to buffer proteostatic collapse upon specific insults. However, whether and how UPR subsystems are coordinated under normal physiological condition, and what is the significance of such coordination might be for aging, remain unknown.

In natural environments where energy resources are often limited, organisms cannot sustain all cellular processes at maximal capacity. This necessitates trade‐offs, where processes that confer the greatest fitness advantage are maintained, while some others are suppressed (Garland 2014). Given the substantial energetic cost of protein folding (Baldwin 2007), it is logical to postulate that similar trade‐offs and coordinated regulation must exist between compartmental UPR systems to optimize resource use. Despite its potential importance, the existence and regulatory principles of such a basal, physiologically programmed trade‐off remains largely unexplored.

The ER faces the most substantial folding burden. It must process proteins with complex topologies, such as transmembrane proteins requiring disulfide bond formation and precise insertion (Brodsky and Skach 2011; Braakman and Hebert 2013; Hong et al. 2022). Consequently, the UPR^ER^ needs to maintain a relatively high basal activity to handle this constitutive demand, which is particularly critical during development characterized by high levels of protein synthesis. This explains why mutations or loss of UPR^ER^ genes severely impair development across multiple species (Mitra and Ryoo 2019). In contrast, a large fraction of cytosolic proteins is structurally simpler and may present a lower constitutive folding burden (Buchberger et al. 2010). These fundamental differences suggest that cells may constantly maintain a high UPR^ER^ activity while keeping basal UPR^cyto^ activity low. Does a specific mechanism exist to enforce this trade‐off? Furthermore, given the pro‐longevity roles of both pathways, a critical question arises: how does the natural, evolutionarily‐set balance between UPR^ER^ and UPR^cyto^ impact the aging process?

Here, we address these questions by identifying the transcription factor LET‐607, the *C. elegans* ortholog of mammalian CREBH, as a key orchestrator of proteostatic balance. While LET‐607 was previously reported to activate UPR^ER^ by us and other laboratories (Weicksel et al. 2016; He et al. 2021), we now demonstrate that it simultaneously represses UPR^cyto^. This reciprocal regulation establishes a trade‐off, favoring UPR^ER^ at the expense of UPR^cyto^ under wild‐type (WT) conditions. Strikingly, deficiency of LET‐607 releases this trade‐off, creating a new, seesaw‐like balance characterized by decreased UPR^ER^ but elevated UPR^cyto^ activity. This rebalancing extends organismal lifespan in a UPR^cyto^‐dependent manner. At the molecular level, we delineate a pathway whereby LET‐607 supports one‐carbon metabolism (1CC) to fuel S‐adenosylmethionine (SAMe) production, which is critical for sustaining H3K9me3‐mediated epigenetic silencing of cytosolic chaperone genes. These findings provide mechanistic insight into how proteostatic trade‐offs are physiologically enforced and suggest that the evolutionarily acquired UPR balance is not optimized for healthy aging, consistent with the antagonistic pleiotropy theory of aging.

## 2 Results and Discussion

### 2.1 WT Animals Maintain UPR^ER^ and UPR^cyto^ Activities in a Reciprocal Manner

**FIGURE 1:**
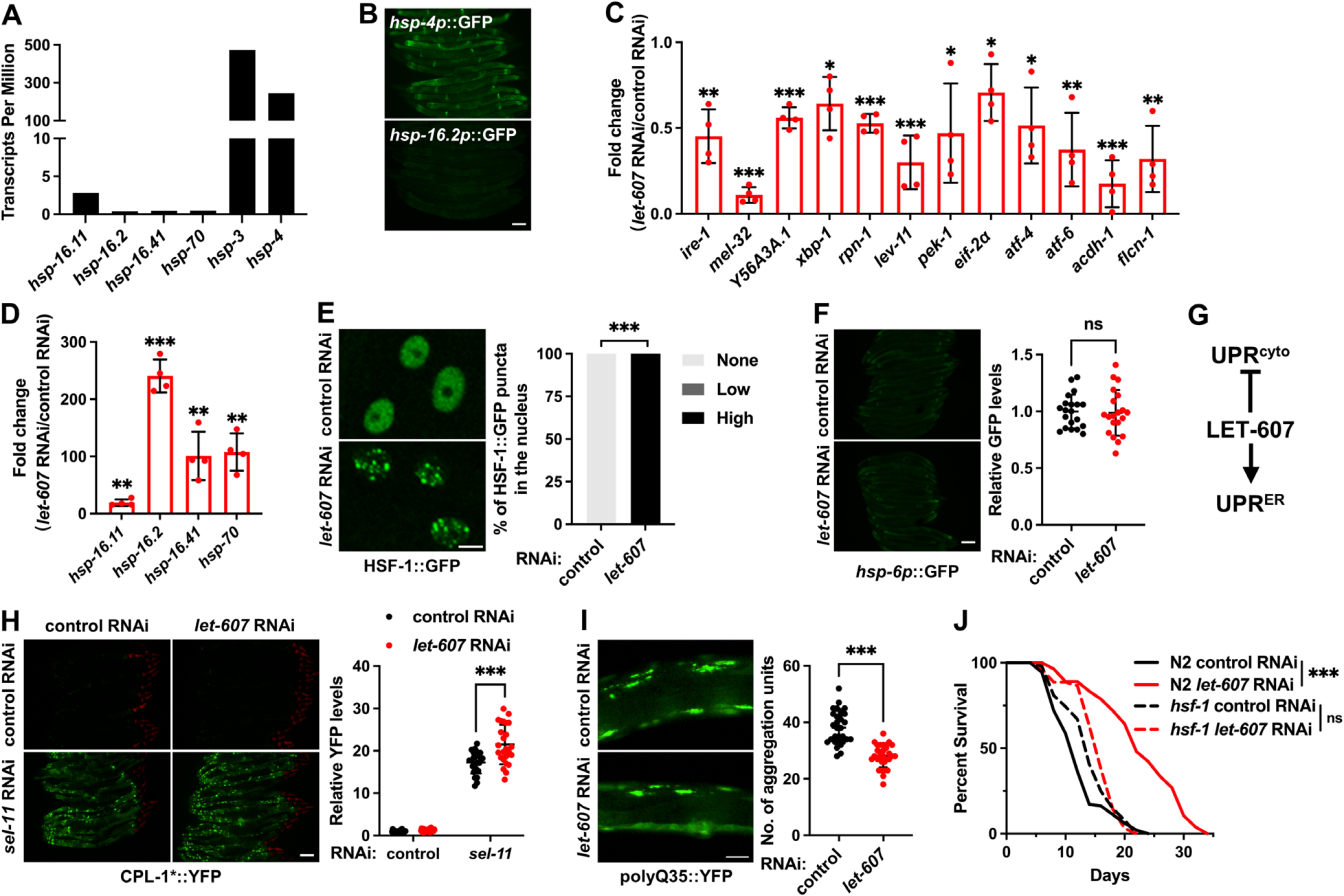
LET‐607 regulates UPR^ER^ and UPR^cyto^ activities in a reciprocal manner. (A) TPM values of ER and cytosolic *hsp* genes in WT Day 1 adult worms based on our published RNA‐seq data (GSE155935). Data represent mean TPM values from three biological replicates. (B) Representative fluorescence images of *hsp‐4p::gfp* and *hsp‐16.2p::gfp* reporters in day 1 adult worms. (C, D) Effect of *let‐607* RNAi on the mRNA levels of representative constitutive‐UPRᴱᴿ genes (C) and cytosolic *hsp* genes (D) in day 1 adult worms. *n* = 4 independent experiments. (E) Effect of *let‐607* RNAi on nuclear accumulation of HSF‐1::GFP in L4 stage worms. HSF‐1::GFP signals in intestinal nuclei were categorized as none, low (1–10 nuclear puncta), or high (> 10 puncta). *n* = 20 animals. (F) Effect of *let‐607* RNAi on *hsp‐6p::gfp* expression in day 1 adult worms. *n* = 20 animals. (G) Schematic diagram illustrating the reciprocal regulation of UPRᴱᴿ and UPRᶜʸᵗ° by LET‐607. (H) Effect of *let‐607* RNAi on CPL‐1^W32A;Y35A^::YFP expression in day 1 adult worms in response to ERAD induction (*sel‐11* RNAi). *n* = 25 animals. (I) Effect of *let‐607* RNAi on polyQ35::YFP foci in day 5 adult worms. *n* = 30 animals. (J) Effect of *hsf‐1(sy441)* mutation on the lifespan of WT and *let‐607(RNAi)* worms. Data are presented as mean ± SD. *p < 0.05, **p < 0.01, ***p < 0.001. Scale bars = 200 μm for (B, F, H), 5 μm for (E), 50 μm for (I). (C–D) were analyzed by multiple *t* tests with Holm–Šidák correction for multiple comparisons. (E) was analyzed by Chi‐square and Fisher's exact test. (F, I) were analyzed by unpaired two‐tailed *t*‐test. (H) was analyzed by two‐way ANOVA with Tukey's multiple comparisons test. (J) was analyzed using the log‐rank (Mantel–Cox) test. Additional biological replicates and full statistical details for lifespan assays are provided in Table S1.

The key effectors of both UPR^ER^ and UPR^cyto^ include compartment‐specific HSPs, whose expression is primarily regulated at the transcriptional level. To assess the relative activities of the UPR^ER^ and UPR^cyto^, we first compared the basal transcriptional levels of these HSPs in WT worms. We utilized our previously published transcriptome data from WT worms (He et al. 2021) and compared the TPM (Transcripts Per Million) values of various HSPs, which allows comparison of different genes within the same sample. The results showed that the expression of characteristic ER HSPs (*hsp‐3*, *hsp‐4*) was several hundred‐fold higher than that of cytosolic HSPs (*hsp‐16.11*, *hsp‐16.2*, *hsp‐16.41*, and *hsp‐16.48*), which exhibited extremely low TPM values (Figure 1A). Consistently, transcriptional reporters showed readily detectable fluorescence from *hsp‐4p::gfp* (UPR^ER^) but not from *hsp‐16.2p::gfp* (UPR^cyto^) under identical imaging conditions (Figure 1B). These results indicate that UPR^ER^ maintains a relatively high basal activity in WT animals, consistent with the substantial protein‐folding burden in the ER, whereas UPR^cyto^ is maintained at a low basal level.

### 2.2 LET‐607 Enforces a Trade‐Off Between UPR^ER^ and UPR^cyto^

The activities of UPR^ER^ and UPR^cyto^ may be regulated separately or in an orchestrated manner. We reasoned that the reciprocal activities of UPR^ER^ and UPR^cyto^ might be coordinated to ensure efficient resource allocation. We therefore hypothesized that a specific regulator exists to enforce this trade‐off by simultaneously activating UPR^ER^ and suppressing UPR^cyto^.

The transcription factor LET‐607, the *C. elegans* homolog of the CREBH, is a known positive regulator of UPR^ER^. It directly binds and regulates UPR^ER^ genes such as *hsp‐3* and *hsp‐4* (Weicksel et al. 2016). Our previous study also showed that *let‐607* RNAi attenuates UPR^ER^ induction upon ER stress (He et al. 2021). We further used qPCR to confirm that knocking down *let‐607* by RNAi (Figure S1A) downregulated multiple constitutive UPR^ER^ genes (c‐UPR) (Figure 1C), whose basal expression requires UPR sensors like IRE‐1/XBP‐1 (Shen et al. 2005).

Our earlier work also revealed that *let‐607* RNAi activates the UPR^cyto^ reporter *hsp‐16.2p::gfp*, suggesting that LET‐607 might be a negative regulator of UPR^cyto^. To confirm this activation of UPR^cyto^, we performed qPCR analysis on several cytosolic HSP genes and found that they were significantly upregulated (Figure 1D). In addition, HSF‐1, the transcription factor regulating UPR^cyto^, was activated, as indicated by an increased formation of nuclear puncta of HSF‐1::GFP (Morton and Lamitina 2013; Deonarine et al. 2021) in *let‐607(RNAi)* animals (Figure 1E). These results demonstrate that LET‐607 functions as a negative regulator of UPR^cyto^. In addition, *let‐607* RNAi did not alter the expression of the mitochondrial UPR reporter *hsp‐6p::gfp* (Figure 1F), suggesting that LET‐607 depletion does not broadly regulate UPR pathways. Taken together, we propose that LET‐607 is a key molecular switch that orchestrates a trade‐off, promoting UPR^ER^ while suppressing UPR^cyto^ (Figure 1G).

### 2.3 UPR Rebalancing Promotes Healthy Aging

It should be noted that full *let‐607* RNAi caused developmental arrest and even diluted *let‐607* RNAi used in this study caused slower development (Figure S1B), suggesting that loss of *let‐607* is evolutionally detrimental. This observation is consistent with the negative impact of UPRᴱᴿ deficiency on animal development. However, since aging itself is not under direct evolutionary selection, and given our previous finding that LET‐607 deficiency extends lifespan (He et al. 2021), we hypothesized that while the UPR rebalancing caused by *let‐607* loss is evolutionarily disadvantageous, it could be beneficial for healthy aging.

To assess the functional consequences of this rebalance, we first examined proteostasis in the ER and cytosol. ER proteostasis was monitored using CPL‐1^W32A;Y35A^::YFP, a misfolded mutant of lysosomal cathepsin L that accumulates in the ER and is subjected to ER‐associated degradation (ERAD) (Miedel et al. 2012). Cytosolic proteostasis was assessed with polyQ35::YFP, which forms aggregates during aging (Morley et al. 2002). We first confirmed that CPL‐1^W32A;Y35A^::YFP responds to reduced UPRᴱᴿ function. RNAi of *xbp‐1*, a key UPRᴱᴿ gene, significantly increased the accumulation of CPL‐1^W32A;Y35A^::YFP in the ER (Figure S1C). Consistent with the conclusion that *let‐607* RNAi suppresses UPRᴱᴿ, *let‐607* RNAi treatment enhanced the accumulation of this reporter under ERAD induction (Figure 1H), with efficient knockdown maintained in double RNAi condition (Figure S1D). Conversely, *let‐607* RNAi significantly reduced the aggregation of polyQ35::YFP during aging (Figure 1I). These results indicate that LET‐607 oppositely modulates proteostasis in the ER and cytosol.

We next directly examined whether this reciprocal regulation of UPR is beneficial for longevity. The results showed that the lifespan extension induced by *let‐607* RNAi was completely abolished by an *hsf‐1* mutation (Figure 1J). In addition, post‐developmental *let‐607* RNAi initiated at the young adult stage still robustly induced *hsp‐16.2p::gfp* expression and extended lifespan (Figure S1E,F), indicating that the beneficial effects of LET‐607 depletion on UPR^cyto^ are not dependent on developmental perturbation. Taken together, these data indicate that, at least in terms of lifespan regulation, activation of UPR^cyto^ outweighs the reduction in UPRᴱᴿ activity, resulting in a net gain in longevity. This also suggests that the evolutionarily optimized UPR balance (WT animals) is suboptimal for longevity, aligning with the concept of antagonistic pleiotropy.

### 2.4 LET‐607 Represses UPR^cyto^ via 1CC

**FIGURE 2:**
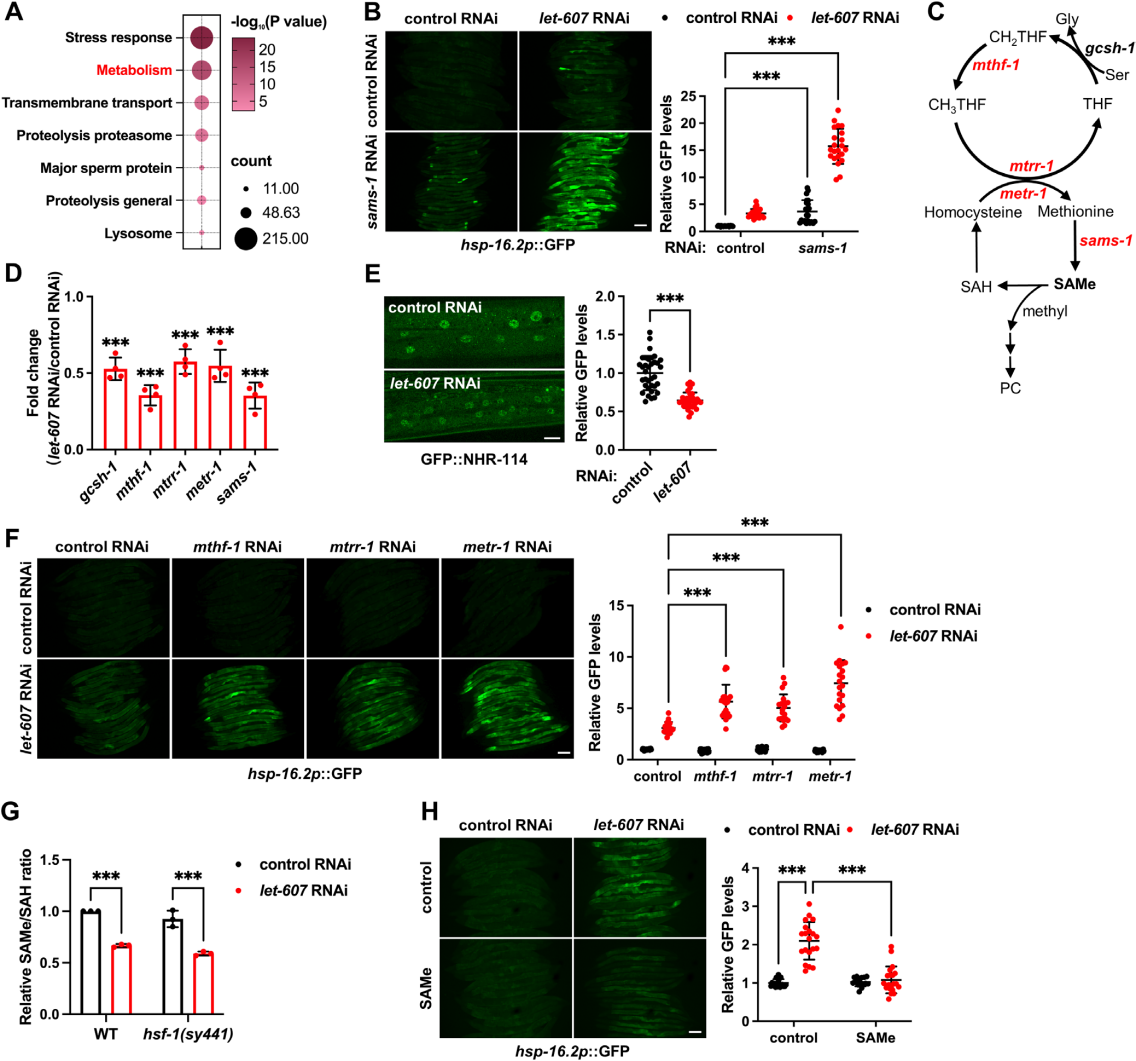
LET‐607 regulates UPRᶜʸᵗ° through the 1CC. (A) Functional enrichment analysis of differentially expressed genes from *let‐607(RNAi)* day 1 adult worms. (B) Effect of *sams‐1* RNAi on *hsp‐16.2p::gfp* expression in WT and *let‐607(RNAi)* day 1 adult worms. *n* = 22 animals. (C) Schematic of 1CC. (D) Effect of *let‐607* RNAi on the mRNA levels of 1CC genes in L4 stage worms. *n* = 4 independent experiments. (E) Effect of *let‐607* RNAi on the nuclear levels of NHR‐114::GFP in L4 stage worms. *n* = 35 nucleus. (F) Effect of 1CC gene RNAi on *hsp‐16.2p::gfp* expression in WT and *let‐607(RNAi)* animals at day 1 adult stages. *n* = 20 animals. (G) Effect of *let‐607* RNAi on the SAMe/SAH ratio in WT and *hsf‐1(sy441)* mutant worms at day 1 adult stage. *n* = 3 independent experiments. (H) Effect of SAMe supplementation on *hsp‐16.2p::gfp* expression in *let‐607(RNAi)* animals at day 1 adult stage. *n* = 20 animals. Data are presented as mean ± SD. ***p < 0.001. Scale bars = 200 μm for (B, F, H); 10 μm for (E). (B, F, G, H) were analyzed by two‐way ANOVA with Tukey's multiple comparisons test. (D) was analyzed by multiple *t* tests with Holm–Šidák correction for multiple comparisons. (E) was analyzed by unpaired two‐tailed *t*‐test.

While LET‐607 directly binds UPR^ER^ genes, the mechanism by which its loss upregulates cytosolic HSPs was unknown. There must be an indirect mechanism underlying such regulation. To explore it, we re‐analyzed our *let‐607(RNAi)* transcriptome data (He et al. 2021) using wormcat, a gene enrichment tool specifically for *C. elegans* study (Holdorf et al. 2020). It revealed significant enrichment of differentially expressed genes involved in metabolism (Figure 2A), suggesting that loss of LET‐607 remodels metabolic pathways. This is consistent with the role of mammalian CREBH as a key regulator of metabolism (Nakagawa and Shimano 2018). We therefore hypothesized that metabolic remodeling may mediate UPR^cyto^ activation upon LET‐607 depletion.

We performed an RNAi screen targeting LET‐607‐regulated metabolic genes. This identified that knockdown of *sams‐1* significantly enhanced UPR^cyto^ reporter *hsp‐16.2p::gfp* in *let‐607(RNAi)* animals (Figure 2B). *sams‐1* RNAi alone also modestly activated *hsp‐16.2p::gfp* (Figure 2B). SAMS‐1 is a key enzyme in the 1CC responsible for generating the methyl donor SAMe (Figure 2C). We speculated that *let‐607* RNAi may regulate the expression of 1CC genes. qPCR analysis confirmed that all tested 1CC genes, including *sams‐1*, were significantly repressed by *let‐607* RNAi (Figure 2D). Furthermore, the nuclear occupancy of NHR‐114, a key transcriptional regulator of 1CC genes, was inhibited by *let‐607* RNAi (Figure 2E). Re‐analysis of published LET‐607 ChIP‐seq datasets (Weicksel et al. 2016) revealed enrichment of LET‐607 occupancy at the promoter regions of key 1CC genes, including *metr‐1* and *mtrr‐1* (Figure S2A), suggesting potential direct transcriptional regulation. Notably, this analysis did not detect LET‐607 binding at the *nhr‐114* promoter, suggesting that LET‐607 deficiency may also indirectly influence 1CC gene expression by modulating NHR‐114 nuclear occupancy. Intriguingly, our previous study (He et al. 2021) demonstrated that LET‐607 regulates DAF‐16 via lipid metabolism remodeling. As a nuclear receptor, NHR‐114 might respond to these metabolic cues to regulate the 1CC pathway, which needs further investigation.

To test whether the 1CC mediates UPR^cyto^ activation upon LET‐607 depletion, we knocked down multiple 1CC genes using RNAi and found that these treatments strongly enhanced *let‐607* RNAi‐induced activation of *hsp‐16.2p::gfp* (Figure 2F), supporting a crucial role for the 1CC in this process. Importantly, similar enhancement was also observed when both *let‐607* and 1CC genes were knocked down specifically during adulthood (Figure S2B), indicating that the interaction between LET‐607 depletion and 1CC inhibition is independent of developmental perturbation.

The 1CC contributes to the production of multiple metabolites. However, since multiple 1CC gene deficiencies enhance *hsp‐16.2p::gfp* in *let‐607(RNAi)* animals (Figure 2F), we reasoned that an end metabolite of 1CC may be responsible for this regulation. Given the role of SAMe as such a metabolite and the observation that only *sams‐1* RNAi alone induces *hsp‐16.2p::gfp*, we hypothesized that SAMe may mediate the effect. Notably, *let‐607* RNAi significantly decreased both SAMe levels and the SAMe/SAH ratio in WT animals, and a similar reduction was also observed in the *hsf‐1(sy441)* mutant background (Figure 2G and Figure S2C). As a positive control, a comparable reduction was also detected upon *sams‐1* RNAi treatment (Figure S2D). These results indicate reduced cellular methylation potential upon LET‐607 depletion independently of HSF‐1. Furthermore, supplementation with exogenous SAMe significantly attenuated *hsp‐16.2p::gfp* induction in *let‐607(RNAi)* animals (Figure 2H). Together, these results suggest that LET‐607 deficiency activates UPR^cyto^ through suppression of 1CC‐dependent SAMe production.

Since SAMe participates in multiple downstream metabolic pathways, we next asked which SAMe‐dependent process mediates UPR^cyto^ activation. One major function of SAMe is to support the synthesis of the membrane lipid phosphatidylcholine (PC) (Figure 2C). However, supplementation with choline, a precursor of PC, failed to suppress *let‐607* RNAi‐induced *hsp‐16.2p::gfp* activation (Figure S2E). Moreover, choline supplementation even enhanced the induction of *hsp‐16.2p::gfp* by *sams‐1* RNAi (Figure S2F). These results suggest that PC is unlikely to mediate the activation of UPR^cyto^ upon LET‐607 depletion.

### 2.5 LIN‐61 Mediates UPR^cyto^ Activation Upon LET‐607 Deficiency

**FIGURE 3:**
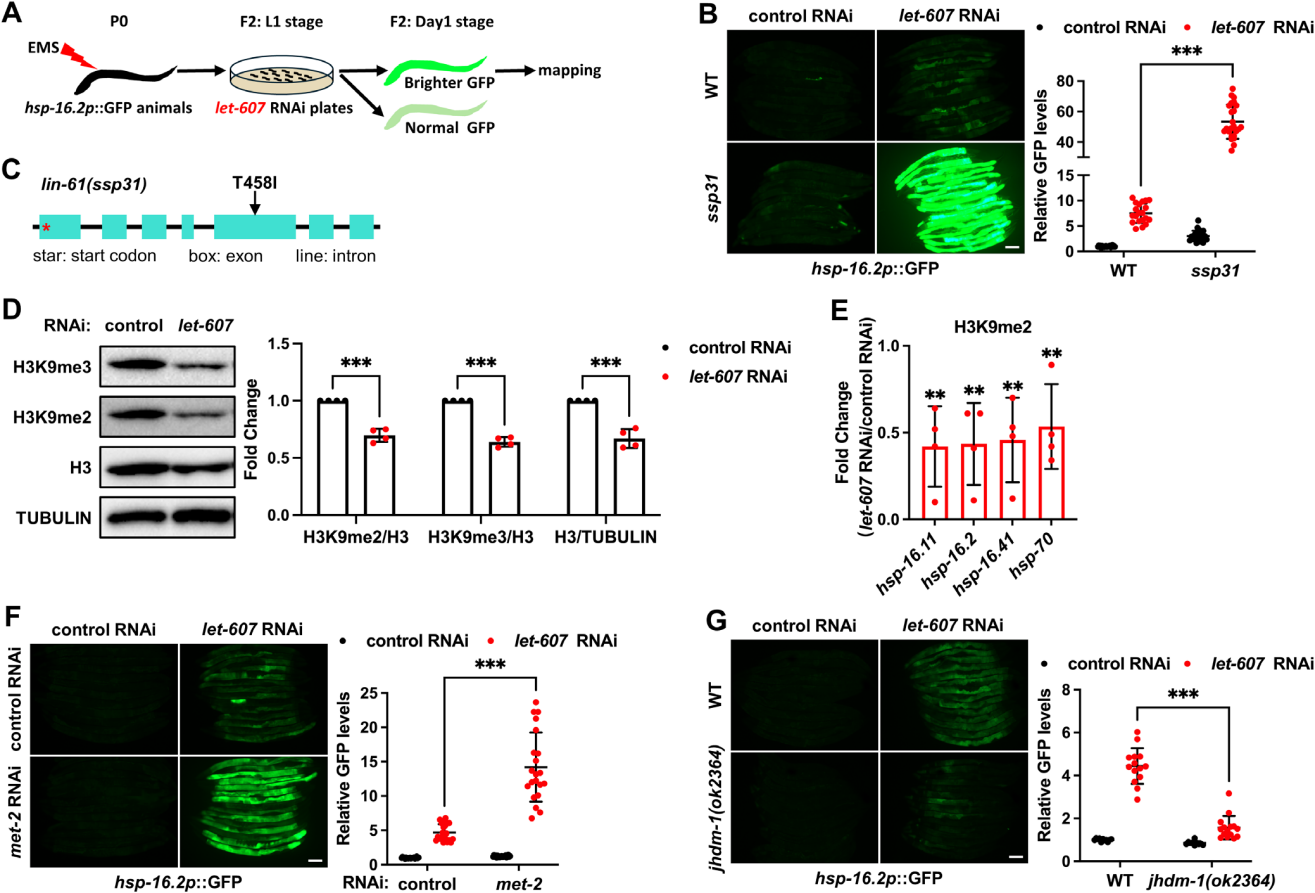
H3K9 methylation links LET‐607 to cytosolic UPR regulation. (A) Schematic of EMS mutagenesis screen to isolate mutants affecting *hsp‐16.2p::gfp* expression under *let‐607(RNAi)*. (B) Effect of *ssp31* mutation on *hsp‐16.2p::gfp* expression in WT and *let‐607(RNAi)* animals at day 1 adult stages. *n* = 20–24 animals. (C) Genetic mapping of *lin‐61(ssp31)* mutation. (D) Western blot analysis of H3K9me2 and H3K9me3 levels in WT and *let‐607(RNAi)* day 1 adult worms. *n* = 4 independent experiments. (E) ChIP‐qPCR analysis of H3K9me2 enrichment at cytosolic *hsp* gene promoters in response to *let‐607* RNAi. *n* = 4 independent experiments. (F, G) Effect of *met‐2(RNAi)* (F) or *jhdm‐1* mutation (G) on *hsp‐16.2p::gfp* expression in WT and *let‐607(RNAi)* animals at day 1 adult stages. *n* = 10–20 animals. Data are presented as mean ± SD. **p < 0.01, ***p < 0.001. Scale bars = 200 μm. (B, F, G) were analyzed by two‐way ANOVA with Tukey's multiple comparisons test. (D, E) was analyzed by multiple *t*‐tests with Holm–Šidák correction for multiple comparisons.

In parallel to conducting the RNAi screen, we also performed an ethyl methane sulfonate (EMS)‐induced forward genetic screen (Figure 3A). This screen identified a recessive mutation *ssp31* that strongly activated the expression of *hsp‐16.2p::gfp* in *let‐607(RNAi)* worms (Figure 3B). We identified the causative mutation in *lin‐61* through SNP‐based mapping and whole‐genome sequencing (Figure 3C, Figure S3A,B), and reintroduction of *lin‐61* expression successfully rescued the mutant phenotype (Figure S3C). Furthermore, both the EMS‐derived *lin‐61(ssp31)* allele and an independent commercially available *lin‐61(n3809)* null mutant (Q159Stop) (Harrison et al. 2007) comparably enhanced *hsp‐16.2p::gfp* expression in *let‐607(RNAi)* animals (Figure S3D), further demonstrating that *lin‐61* mediates UPR^cyto^ activation upon LET‐607 deficiency.

LIN‐61 is an MBT domain protein that recognizes methylation of histone H3 lysine 9 and interacts with HPL‐2 and LIN‐13 to form a complex essential for maintaining the chromatin‐suppressive state associated with H3K9me (Koester‐Eiserfunke and Fischle 2011; Wu et al. 2012). The EMS‐derived *lin‐61(ssp31)* allele carries a missense mutation within the fourth MBT domain, which is important for recognition of H3K9me‐marked chromatin. Notably, both *lin‐61(ssp31)* and *lin‐61(n3809)* mutants exhibited comparable reductions in global H3K9me2 and H3K9me3 levels (Figure S3E), supporting impaired H3K9me‐associated chromatin repression in these mutants. Consistent with this role, RNAi knockdown of *lin‐61*, *lin‐13*, and *hpl‐2* all enhanced *hsp‐16.2p::gfp* expression in *let‐607(RNAi)* animals (Figure S3F), suggesting that de‐repression of H3K9me contributes to UPR^cyto^ following LET‐607 deficiency.

The involvement of LIN‐61 aligns with the function of SAMe as a methyl donor in regulating histone methylation. We thus next examined whether LET‐607 influences H3K9me‐associated chromatin repression. Western blot analysis revealed that knockdown of *let‐607* reduced global levels of both H3K9me2 and H3K9me3 (Figure 3D). ChIP‐qPCR analysis confirmed a decrease of H3K9me2 at the promoters of cytosolic HSP genes (Figure 3E and Figure S3G). To functionally link H3K9me2 loss to UPR^cyto^ activation, we manipulated the pathway. In *C. elegans* , MET‐2 is the major histone H3K9 methyltransferase responsible for depositing H3K9me1 and H3K9me2. RNAi against *met‐2* further decreased H3K9me2 levels in *let‐607(RNAi)* animals (Figure S3H), which correspondingly enhanced *hsp‐16.2p::gfp* expression (Figure 3F). Conversely, RNAi targeting *jhdm‐1*, a putative H3K9me2 demethylase, restored H3K9me2 levels in *let‐607(RNAi)* animals (Figure S3H), and mutation of *jhdm‐1* suppressed *hsp‐16.2p::gfp* expression (Figure 3G). Together, these findings suggest that the reduction of repressive H3K9me2 is required for UPR^cyto^ activation in response to LET‐607 deficiency.

### 2.6 H3K9me Removal Sufficiently Induces UPR^cyto^

**FIGURE 4:**
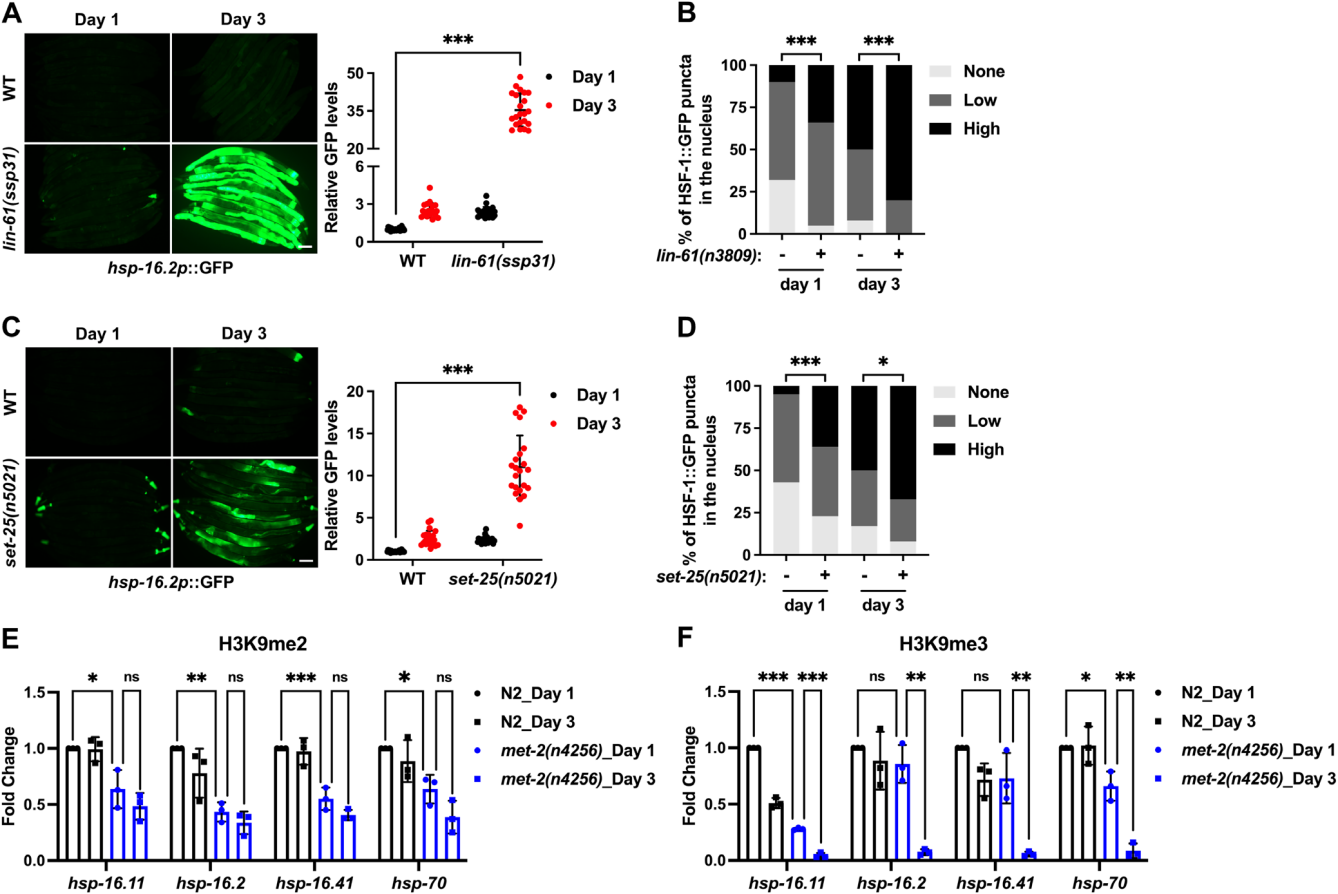
H3K9me3 loss drives UPR^cyto^ activation in aging adults (A, C) Effects of *lin‐61* mutation (A) and *set‐25* mutation (C) on *hsp‐16.2p::gfp* expression in day 1 and day 3 adults. *n* = 22 animals. (B, D) Effects of *lin‐61* mutation (B) and *set‐25* mutation (D) on HSF‐1::GFP nuclear occupancy in day 1 and day 3 adults. *n* = 15–25 animals. (E, F) ChIP‐qPCR analysis of promoter‐associated H3K9me2 (E) and H3K9me3 (F) levels at cytosolic *hsp* gene loci in WT and *met‐2(n4256)* worms at day 1 and day 3 adult stages. *n* = 3 independent experiments. Data are presented as mean ± SD. *p < 0.05, **p < 0.01, ***p < 0.001. Scale bars = 200 μm. (A, C, E, F) were analyzed by two‐way ANOVA with Tukey's multiple comparisons test. (B, D) were analyzed by chi‐square and Fisher's exact test.

We noted that even in the absence of *let‐607* RNAi, the *lin‐61* mutants could modestly induce the expression of *hsp‐16.2p::gfp* (Figure 3B). We asked whether this induction might strengthen with age. The results showed that *lin‐61* mutation only weakly activated the UPR^cyto^ reporter in day 1 adults, but this activation was markedly stronger by day 3 of adulthood (Figure 4A). A similar phenomenon was observed in *met‐2(RNAi)* worms (Figure S4A). Furthermore, examination of HSF‐1::GFP revealed that both the *lin‐61* and *met‐2* mutations induced more pronounced nuclear accumulation of HSF‐1::GFP puncta with age (Figure 4B and Figure S4B). These results indicate that removal of H3K9me2‐mediated epigenetic repression is sufficient to activate UPR^cyto^ in an age‐dependent manner.

H3K9me2 serves as a precursor to H3K9me3, the latter also being an important repressive histone mark. We thus asked if H3K9me3 also plays a role. Mutation of *set‐25*, which encodes the H3K9 tri‐methyltransferase, not only enhanced UPR^cyto^ activation in *let‐607(RNAi)* animals (Figure S4C) but also autonomously induced *hsp‐16.2p::gfp* expression (Figure 4C) and HSF‐1::GFP puncta formation in day 3 adults (Figure 4D). Thus, both H3K9me2 and H3K9me3 are critical for LET‐607‐mediated repression of UPR^cyto^ genes.

To directly examine age‐dependent changes in H3K9 methylation, we analyzed global levels of H3K9me2 and H3K9me3 in *lin‐61*, *met‐2*, and *set‐25* mutants at day 1 and day 3 adults. Both marks were already substantially reduced by day 1 and did not further decline by day 3 (Figure S4D–F). We next performed ChIP‐qPCR analysis at representative UPR^cyto^ target loci in *met‐2* mutants. H3K9me2 occupancy was significantly reduced at both day 1 and day 3, with no further decrease during aging (Figure 4E). In contrast, H3K9me3 levels at these loci were further reduced in day 3 adults compared to day 1 adults (Figure 4F), suggesting that progressive loss of promoter‐associated H3K9me3 underlies age‐dependent UPR^cyto^ activation.

We next investigated whether LET‐607‐mediated UPR^cyto^ activation is specific to H3K9 methylation by examining additional histone methylation pathways. Depletion of H3K4 methyltransferases modestly enhanced *hsp‐16.2p::gfp* induction in *let‐607(RNAi)* animals, whereas perturbation of H3K36 or H3K27 methylation pathways had no effect (Figure S5A,B). Notably, the enhancement caused by H3K4 methyltransferase depletion was substantially weaker than that observed upon disruption of H3K9me‐associated repression. These results support a predominant and relatively specific role for H3K9me‐associated chromatin repression in suppressing UPR^cyto^ downstream of LET‐607.

### 2.7 H3K9me Loss Enhances Stress Resilience and Promotes Longevity Through UPR^cyto^

The lifespan extension from LET‐607 deficiency suggests that its enforced gene repression program, while beneficial for development, may be suboptimal for aging. Since H3K9me modification is crucial for normal development, with mutations in regulators like *lin‐61* or *set‐25* causing embryonic defects (Padeken et al. 2021). we hypothesized that this chromatin pathway exhibits antagonistic pleiotropy: essential for development but detrimental to longevity.

**FIGURE 5:**
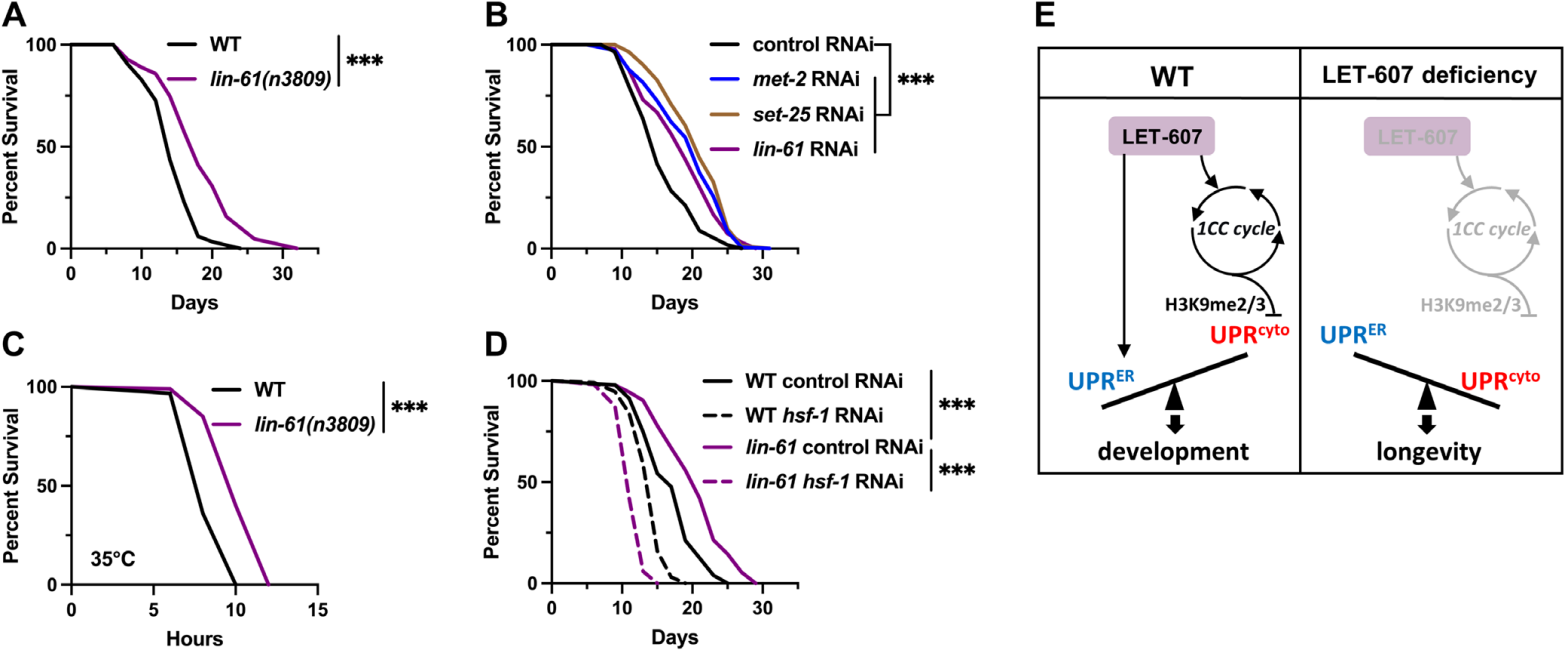
Loss of H3K9 methylation enhances proteostasis and longevity. (A) Effect of *lin‐61* mutation on lifespan. (B) Effect of post‐developmental RNAi targeting *met‐2*, *set‐25* and *lin‐61* RNAi on lifespan. (C) Effect of *lin‐61* mutation on heat stress resistance. (D) Effect of *hsf‐1* RNAi on the lifespan of *lin‐61(n3809)* mutants. (E) Proposed working model: In WT animals, LET‐607 sustains UPR^ER^ activity to support normal development. Simultaneously, it promotes 1CC and H3K9 methylation, which epigenetically repress UPR^cyto^ genes. Conversely, loss of LET‐607 impairs UPRᴱᴿ, while also reducing 1CC activity and H3K9me levels. This leads to derepression of UPR^cyto^ genes, enhanced cytosolic proteostasis, and extends lifespan. Data are presented as mean ± SD. ***p < 0.001. (A‐D) were analyzed by log‐rank (Mantel–Cox) test, the additional repeat assays and the corresponding statistical analyses are provided in Table S1.

To test this hypothesis, we examined animals that reached adulthood despite *lin‐61* mutation. These adults showed significant lifespan extension compared to WT controls (Figure 5A). This longevity benefit was recapitulated in animals subjected to RNAi targeting *lin‐61*, *met‐2*, and *set‐25* (Figure 5B). Moreover, *lin‐61* mutants exhibited enhanced thermotolerance (Figure 5C), and *hsf‐1* RNAi abolished their longevity phenotype (Figure 5D), establishing HSF‐1 as the principal mediator of longevity in *lin‐61* mutants. Collectively, these results suggest that the loss of H3K9me plays a beneficial role in stress resistance and longevity.

This study reveals that the transcription factor LET‐607 functions as a master regulator that enforces a trade‐off between the UPR^ER^ and UPR^cyto^, maintaining a proteostatic balance optimized for developmental fitness but suboptimal for longevity (Figure 5E). We demonstrate that LET‐607 supports 1CC to sustain H3K9 methylation, which epigenetically represses cytosolic chaperone genes. Disruption of LET‐607, or the repressive H3K9me mark it sustains, unlocks UPR^cyto^ activation, enhancing proteostasis in the cytosol and promoting longevity in an HSF‐1–dependent manner.

Interestingly, previous studies reported that *jhdm‐1* deficiency extends lifespan in *C. elegans* (Lee et al. 2019). In contrast, our findings suggest that under conditions requiring elevated UPR^cyto^ activity, such as LET‐607 deficiency, increased H3K9 methylation caused by *jhdm‐1* loss may become detrimental by restricting cytosolic proteostasis. We propose that the physiological consequences of altered H3K9 methylation are highly context‐dependent, varying according to the pre‐existing proteostatic state.

While this UPR trade‐off has been implicated in lifespan regulation in this study, its functional significance in other physiological or pathological conditions remains unclear. Given that mammalian CREBH can be activated by immune and metabolic signals (Zhang et al. 2006), we speculate that its nematode homolog LET‐607 may also respond to environmental challenges, such as pathogen infection or nutritional stress, and thereby modulate the balance between UPR systems. Further investigation of this regulatory mechanism will help elucidate the functional roles of UPR trade‐off under both physiological and pathological conditions.

The antagonistic pleiotropy theory of aging posits that genes contributing to aging are favored by natural selection due to their beneficial effects in early life, even if they have detrimental effects in later life (Williams 1957). Our findings provide an illustration. The UPR balance observed in WT animals (high UPR^ER^, low UPR^cyto^) is evolutionarily advantageous, as it ensures proper development and fitness in early life. However, this same configuration becomes detrimental later in life, as it constrains a latent pro‐longevity pathway mediated by the UPR^cyto^. The fact that both the regulator (LET‐607) and the epigenetic mark (H3K9me) it controls exhibit this pleiotropic pattern, essential for development yet suppressive of longevity, this strongly suggests that evolutionary pressures have actively tuned UPR^cyto^ activity downward, with aging as a trade‐off. We therefore propose that the evolutionary optimization of proteostatic resource allocation is a key factor influencing lifespan.

## 3 Methods

### 3.1 *C. elegans* Strains and Maintenance

*C. elegans* were cultured on standard nematode medium (NGM) seeded with *E. coli* OP50‐1 (Brenner 1974). The following strains were provided by Caenorhabditis Genome Center: wild‐type N2 Bristol and CB4856 Hawaiian, TJ375[*hsp‐16.2p::gfp*], SJ4005[*hsp‐4p::gfp*], OG497[*hsf‐1::gfp*], VK1879[*nhx‐2p::cpl‐1* ^W32A Y35A^::YFP], AM140[*unc‐54p*::Q35::YFP], PS3551[*hsf‐1(sy441)*], MT12833[*lin‐61(n3809)*], MT13293[*met‐2(n4256)*], MT17463[*set‐25(n5021)*], and RB1826[*jhdm‐1(ok2364)*]. The strain *lin‐61(ssp31)* was generated in our laboratory by EMS mutagenesis. WU55 [*gfp::2xflag::nhr‐114*] was kindly provided by Dr. Lianfeng Wu. Double mutants were generated by standard genetic techniques.

### 3.2 Genetic Mapping and Cloning

The *ssp31* mutant was mapped using single nucleotide polymorphism (SNP)‐based mapping as previously described (Davis et al. 2005). Briefly, *ssp31* Bristol strain hermaphrodites carrying the *hsp‐16.2p::gfp* reporter were crossed with CB4856 Hawaiian males. F2 progeny were subjected to *let‐607* RNAi treatment, and individual F2 animals exhibiting markedly enhanced GFP fluorescence were singled and propagated. F2 animals with normal GFP intensity were collected together as controls.

For chromosome mapping, genomic DNA from singled bright F2 animals was pooled together and compared with genomic DNA from the control F2 population. SNP markers distributed across the genome were analyzed to determine linkage to a chromosome. For interval mapping, the same procedure was performed using genomic DNA prepared separately from individual bright F2 recombinants. The *ssp31* mutation was mapped to chromosome I near the −1 SNP marker. Whole‐genome sequencing subsequently identified 27 protein‐altering variants in that region, including 25 nonsynonymous single nucleotide variants and 2 stop‐gain mutations, affecting 15 genes. RNAi targeting these 15 genes were performed and only *lin‐61* RNAi enhanced *let‐607(RNAi)*‐induced *hsp‐16.2p::gfp*.

A rescue construct containing the genomic locus of *lin‐61*, including ~2 kb upstream promoter sequence, was cloned into the pDD95_79 vector and microinjected into *ssp31* animals together with the co‐injection marker *myo‐2p::mCherry* for rescue analysis.

### 3.3 RNA Interference Treatment

For the RNAi experiment, HT115 bacteria containing specific dsRNA‐expression plasmids (Ahringer library) (Kamath et al. 2003) were cultured overnight at 37°C in LB medium supplemented with 100 μg/mL carbenicillin and seeded onto NGM plates containing 5 mM IPTG. Because strong *let‐607* RNAi caused developmental defects, *let‐607* RNAi bacteria were diluted with control RNAi bacteria at a 1:10 ratio in all experiments. For double RNAi experiments, *let‐607* RNAi bacteria were mixed with the indicated RNAi bacteria at a 1:10 ratio; while corresponding single RNAi controls were adjusted with control RNAi bacteria to maintain comparable bacterial concentrations across conditions.

### 3.4 qRT‐PCR

qRT‐PCR was performed as previously described (Wang et al. 2021). Briefly, day 1 adult worms were collected, washed in M9 buffer, and homogenized in Trizol reagent (Life Technologies). RNA was extracted according to the manufacturer's protocol, and DNA contamination was removed using DNase I (Thermo Fisher Scientific). The RNA was then reverse transcribed to cDNA using the RevertAid First Strand cDNA Synthesis Kit (Thermo Fisher Scientific). Quantitative PCR was carried out using SYBR Green (Bio‐Rad), and data were collected with CFX Maestro Software. Primer sequences were listed in Table S2.

### 3.5 Immunoblotting

Day 1 adult worms were collected and sonicated in RIPA buffer containing 1 mM DTT and proteinase inhibitor (Beyotime) before boiling and loading. Antibodies against H3 (Abcam, ab1791, 1:2000), H3K9me2 (Abcam, ab1220, 1:2000), H3K9me3 (Abcam, ab8898, 1:2000), and TUBULIN (Sigma, T9026, 1:2000) were used. The images were quantified by Image J 1.54 m software.

### 3.6 Chromatin Immunoprecipitation (ChIP)

ChIP assays were performed as described (Mukhopadhyay et al. 2008) with modifications. Briefly, ~10,000 synchronized day‐1 adult worms were collected, washed with M9 buffer, and crosslinked in 1% formaldehyde in PBS for 20 min at room temperature with gentle rocking. Crosslinking was quenched with 0.125 M glycine for 15 min. Worm pellets were washed, snap‐frozen in liquid nitrogen, and stored at −80°C.

For chromatin preparation, worms were resuspended in HEPES lysis buffer (50 mM HEPES‐KOH pH 7.5, 150 mM NaCl, 1% Triton X‐100, 0.1% SDS, 1% sodium deoxycholate, 1 mM EDTA, protease inhibitors) and sonicated to obtain DNA fragments of 300–1000 bp. After centrifugation, the supernatant was incubated overnight at 4°C with 2 μg of anti‐H3K9me2 antibody, anti‐H3K9me3 antibody, or the corresponding control IgG/IgG2α, together with pre‐cleared salmon sperm DNA/protein A agarose beads (Millipore). Beads were sequentially washed with low‐salt, high‐salt, and LiCl buffers followed by TE buffer. DNA–protein crosslinks were reversed at 65°C overnight with proteinase K, and DNA was purified by phenol–chloroform extraction and ethanol precipitation. Purified DNA was analyzed by quantitative PCR using promoter‐specific primers for target hsp genes and normalized to input DNA.

### 3.7 Fluorescent Microscopy

To analyze GFP fluorescence, adult worms with indicated stages were paralyzed in 1 mM levamisole and mounted on slides. Images were acquired using Nikon NIS‐Elements or Leica LAS X software. The fluorescence intensity of *hsp‐16.2p::gfp* and *cpl‐1* ^W32A Y35A^::YFP were quantified using ImageJ v1.54m, while GFP::NHR‐114 intensity was analyzed with Leica LAS X software. Protein aggregation in AM140 worms was assessed by measuring the number of polyQ35::YFP foci in each worm. To evaluate HSF‐1 nuclear localization, HSF‐1::GFP signals in intestinal nuclei were categorized as none, low, or high. “Low” indicated nuclei containing 1–10 HSF‐1::GFP puncta, whereas “high” indicated nuclei containing more than 10 puncta.

### 3.8 Choline and SAMe Supplementation

Choline (Sigma, V900442) and S‐Adenosyl‐L‐methionine disulfate tosylate (SAMe; Macklin, S837184) were diluted in M9 buffer and applied onto the surface of the bacterial lawn to final concentrations of 15 mM and 5 mM, respectively. L1 stage animals were subsequently transferred to these plates and incubated for 72 h before further analysis.

### 3.9 Survival Analysis

Lifespan assays were performed as previously described (Feng et al. 2025). Briefly, synchronized L1 worms were added to NGM plates for lifespan analysis at 20°C. The survival rates were scored every other day. Worms that died of vulva burst, bagging or crawling off the plates were censored. For heat shock resistance, day‐1 adults were incubated at 35°C and scored for viability at indicated intervals.

### 3.10 Quantification and Statistical Analysis

Data are presented as mean ± SD. Statistical analysis was performed using GraphPad Prism software. Survival data were analyzed using a log‐rank (Mantel‐Cox) test. Other data were analyzed by using an ANOVA or *t*‐test, as indicated in the figure legends. *p* < 0.05 was considered significant. Micrographic and immunoblotting images are representative of at least three independent experiments with similar results.

## Author Contributions

H.T. (Haiqing Tang) conceived and supervised the study. H.T. (Haixiang Tong), W.L., P.Y., S.P., and H.T. (Haiqing Tang) designed the experiments. H.T. (Haixiang Tong), W.L., P.Y., F.L., and Q.L. performed the experiments. H.T. (Haiqing Tang) and S.P. wrote the original draft of the manuscript. H.T. (Haixiang Tong), S.P., and H.T. (Haiqing Tang) edited the manuscript. S.P. and H.T. (Haiqing Tang) acquired funding.

## Funding

This work was supported by the National Natural Science Foundation of China (Grant No. 32370828 [to H.T.] and Grant No. 32571349 [to S.P.]).

## Conflicts of Interest

The authors declare no conflicts of interest.

## Supporting information


**Figure S1:** Additional characterization of LET‐607 depletion and UPR^cyto^ activation.
**Figure S2:** SAMe metabolism mediates UPR^cyto^ regulation upon LET‐607 deficiency.
**Figure S3:** Identification of *lin‐61* and H3K9me‐associated chromatin factors in the regulation of UPR^cyto^.
**Figure S4:** H3K9me loss enhances UPR^cyto^ activation.
**Figure S5:** H3K9 methylation plays a predominant role in the regulation of UPR^cyto^.
**Table S1:** Survival data. Repeats 1 were graphed in figures.
**Table S2:** qPCR primer sequences.

## Data Availability

All data are contained within the article.

## References

[acel70620-bib-0001] Acosta‐Alvear, D. , J. M. Harnoss , P. Walter , and A. Ashkenazi . 2025. “Homeostasis Control in Health and Disease by the Unfolded Protein Response.” Nature Reviews. Molecular Cell Biology 26: 193–212. 10.1038/s41580-024-00794-0. 39501044

[acel70620-bib-0002] Baldwin, R. L. 2007. “Energetics of Protein Folding.” Journal of Molecular Biology 371: 283–301. 10.1016/j.jmb.2007.05.078. 17582437

[acel70620-bib-0003] Braakman, I. , and D. N. Hebert . 2013. “Protein Folding in the Endoplasmic Reticulum.” Cold Spring Harbor Perspectives in Biology 5: a013201. 10.1101/cshperspect.a013201. 23637286 PMC3632058

[acel70620-bib-0004] Brenner, S. 1974. “The Genetics of *Caenorhabditis elegans* .” Genetics 77: 71–94. 10.1093/genetics/77.1.71. 4366476 PMC1213120

[acel70620-bib-0005] Brodsky, J. L. , and W. R. Skach . 2011. “Protein Folding and Quality Control in the Endoplasmic Reticulum: Recent Lessons From Yeast and Mammalian Cell Systems.” Current Opinion in Cell Biology 23: 464–475. 10.1016/j.ceb.2011.05.004. 21664808 PMC3154734

[acel70620-bib-0006] Buchberger, A. , B. Bukau , and T. Sommer . 2010. “Protein Quality Control in the Cytosol and the Endoplasmic Reticulum: Brothers in Arms.” Molecular Cell 40: 238–252. 10.1016/j.molcel.2010.10.001. 20965419

[acel70620-bib-0007] Davis, M. W. , M. Hammarlund , T. Harrach , P. Hullett , S. Olsen , and E. M. Jorgensen . 2005. “Rapid Single Nucleotide Polymorphism Mapping in *C. elegans* .” BMC Genomics 6: 118. 10.1186/1471-2164-6-118. 16156901 PMC1242227

[acel70620-bib-0008] Deonarine, A. , M. W. G. Walker , and S. D. Westerheide . 2021. “HSF‐1 Displays Nuclear Stress Body Formation in Multiple Tissues in *Caenorhabditis elegans* Upon Stress and Following the Transition to Adulthood.” Cell Stress & Chaperones 26: 417–431. 10.1007/s12192-020-01188-9. 33392968 PMC7925714

[acel70620-bib-0009] De‐Souza, E. A. , M. A. Thompson , and R. C. Taylor . 2023. “Olfactory Chemosensation Extends Lifespan Through TGF‐β Signaling and UPR Activation.” Nature Aging 3: 938–947. 10.1038/s43587-023-00467-1. 37500972 PMC10432268

[acel70620-bib-0010] Durieux, J. , S. Wolff , and A. Dillin . 2011. “The Cell‐Non‐Autonomous Nature of Electron Transport Chain‐Mediated Longevity.” Cell 144: 79–91. 10.1016/j.cell.2010.12.016. 21215371 PMC3062502

[acel70620-bib-0011] Feng, X. , X. Wang , S. Guang , S. Pang , and H. Tang . 2025. “Inhibition of the Nucleolar RNA Exosome Facilitates Adaptation to Starvation.” PLoS Biology 23: e3003190. 10.1371/journal.pbio.3003190. 40397874 PMC12136472

[acel70620-bib-0012] Garland, T. 2014. “Trade‐Offs.” Current Biology 24: R60–R61. 10.1016/j.cub.2013.11.036. 24456973

[acel70620-bib-0013] Harrison, M. M. , X. Lu , and H. R. Horvitz . 2007. “LIN‐61, One of Two *Caenorhabditis elegans* Malignant‐Brain‐Tumor‐Repeat‐ Containing Proteins, Acts With the DRM and NuRD‐Like Protein Complexes in Vulval Development but Not in Certain Other Biological Processes.” Genetics 176: 255–271. 10.1534/genetics.106.069633. 17409073 PMC1893064

[acel70620-bib-0014] He, B. , J. Xu , S. Pang , and H. Tang . 2021. “Phosphatidylcholine Mediates the Crosstalk Between LET‐607 and DAF‐16 Stress Response Pathways.” PLoS Genetics 17: e1009573. 10.1371/journal.pgen.1009573. 34014977 PMC8172019

[acel70620-bib-0015] Hetz, C. , K. Zhang , and R. J. Kaufman . 2020. “Mechanisms, Regulation and Functions of the Unfolded Protein Response.” Nature Reviews. Molecular Cell Biology 21: 421–438. 10.1038/s41580-020-0250-z. 32457508 PMC8867924

[acel70620-bib-0016] Hipp, M. S. , P. Kasturi , and F. U. Hartl . 2019. “The Proteostasis Network and Its Decline in Ageing.” Nature Reviews. Molecular Cell Biology 20: 421–435. 10.1038/s41580-019-0101-y. 30733602

[acel70620-bib-0017] Holdorf, A. D. , D. P. Higgins , A. C. Hart , et al. 2020. “WormCat: An Online Tool for Annotation and Visualization of *Caenorhabditis elegans* Genome‐Scale Data.” Genetics 214: 279–294. 10.1534/genetics.119.302919. 31810987 PMC7017019

[acel70620-bib-0018] Hong, H. , H. K. Choi , and T. Y. Yoon . 2022. “Untangling the Complexity of Membrane Protein Folding.” Current Opinion in Structural Biology 72: 237–247. 10.1016/j.sbi.2021.11.013. 34995926 PMC9476714

[acel70620-bib-0019] Hsu, A. L. , C. T. Murphy , and C. Kenyon . 2003. “Regulation of Aging and Age‐Related Disease by DAF‐16 and Heat‐Shock Factor.” Science 300: 1142–1145. 10.1126/science.1083701. 12750521

[acel70620-bib-0020] Imanikia, S. , M. Sheng , C. Castro , J. L. Griffin , and R. C. Taylor . 2019. “XBP‐1 Remodels Lipid Metabolism to Extend Longevity.” Cell Reports 28: 581–589. 10.1016/j.celrep.2019.06.057. 31315038 PMC6656787

[acel70620-bib-0021] Kamath, R. S. , A. G. Fraser , Y. Dong , et al. 2003. “Systematic Functional Analysis of the *Caenorhabditis elegans* Genome Using RNAi.” Nature 421: 231–237. 10.1038/nature01278. 12529635

[acel70620-bib-0022] Kim, H. , A. R. Grant , M. S. Simic , et al. 2016. “Lipid Biosynthesis Coordinates a Mitochondrial‐To‐ Cytosolic Stress Response.” Cell 166: 1539–1552. 10.1016/j.cell.2016.08.027. 27610574 PMC5922983

[acel70620-bib-0023] Klaips, C. L. , G. G. Jayaraj , and F. U. Hartl . 2018. “Pathways of Cellular Proteostasis in Aging and Disease.” Journal of Cell Biology 217: 51–63. 10.1083/jcb.201709072. 29127110 PMC5748993

[acel70620-bib-0024] Koester‐Eiserfunke, N. , and W. Fischle . 2011. “H3K9me2/3 Binding of the MBT Domain Protein LIN‐61 Is Essential for *Caenorhabditis elegans* Vulva Development.” PLoS Genetics 7: e1002017. 10.1371/journal.pgen.1002017. 21437264 PMC3060068

[acel70620-bib-0025] Lee, T. W. , H. S. David , A. K. Engstrom , B. S. Carpenter , and D. J. Katz . 2019. “Repressive H3K9me2 Protects Lifespan Against the Transgenerational Burden of COMPASS Activity in *C. elegans* .” eLife 8: e48498. 10.7554/eLife.48498. 31815663 PMC7299346

[acel70620-bib-0026] Li, D. , D. Chen , W. Li , and G. Ou . 2024. “Inhibition of a Cyclic Nucleotide‐ Gated Channel on Neuronal Cilia Activates Unfolded Protein Response in Intestinal Cells to Promote Longevity.” Proceedings of the National Academy of Sciences of the United States of America 121: e2321228121. 10.1073/pnas.2321228121. 38857399 PMC11194586

[acel70620-bib-0027] Li, J. J. , N. Xin , C. Yang , et al. 2025. “Unveiling the Intercompartmental Signaling Axis: Mitochondrial to ER Stress Response (MERSR) and Its Impact on Proteostasis.” PLoS Genetics 21: e1011700. 10.1371/journal.pgen.1011700. 40338975 PMC12088515

[acel70620-bib-0028] López‐Otín, C. , M. A. Blasco , L. Partridge , M. Serrano , and G. Kroemer . 2023. “Hallmarks of Aging: An Expanding Universe.” Cell 186: 243–278. 10.1016/j.cell.2022.11.001. 36599349

[acel70620-bib-0029] Miedel, M. T. , N. J. Graf , K. E. Stephen , et al. 2012. “A Pro‐Cathepsin L Mutant Is a Luminal Substrate for Endoplasmic‐Reticulum‐Associated Degradation in *C. elegans* .” PLoS One 7: e40145. 10.1371/journal.pone.0040145. 22768338 PMC3388072

[acel70620-bib-0030] Mitra, S. , and H. D. Ryoo . 2019. “The Unfolded Protein Response in Metazoan Development.” Journal of Cell Science 132: jcs217216. 10.1242/jcs.217216. 30770479 PMC6432711

[acel70620-bib-0031] Morley, J. F. , H. R. Brignull , J. J. Weyers , and R. I. Morimoto . 2002. “The Threshold for Polyglutamine‐Expansion Protein Aggregation and Cellular Toxicity Is Dynamic and Influenced by Aging in *Caenorhabditis elegans* .” Proceedings of the National Academy of Sciences of the United States of America 99: 10417–10422. 10.1073/pnas.152161099. 12122205 PMC124929

[acel70620-bib-0032] Morton, E. A. , and T. Lamitina . 2013. “ *Caenorhabditis elegans* HSF‐1 Is an Essential Nuclear Protein That Forms Stress Granule‐Like Structures Following Heat Shock.” Aging Cell 12: 112–120. 10.1111/acel.12024. 23107491 PMC3552056

[acel70620-bib-0033] Mukhopadhyay, A. , B. Deplancke , A. J. Walhout , and H. A. Tissenbaum . 2008. “Chromatin Immunoprecipitation (ChIP) Coupled to Detection by Quantitative Real‐Time PCR to Study Transcription Factor Binding to DNA in *Caenorhabditis elegans* .” Nature Protocols 3: 698–709. 10.1038/nprot.2008.38. 18388953 PMC2681100

[acel70620-bib-0034] Nakagawa, Y. , and H. Shimano . 2018. “CREBH Regulates Systemic Glucose and Lipid Metabolism.” International Journal of Molecular Sciences 19: 1396. 10.3390/ijms19051396. 29738435 PMC5983805

[acel70620-bib-0035] Padeken, J. , S. Methot , P. Zeller , C. E. Delaney , V. Kalck , and S. M. Gasser . 2021. “Argonaute NRDE‐3 and MBT Domain Protein LIN‐61 Redundantly Recruit an H3K9me3 HMT to Prevent Embryonic Lethality and Transposon Expression.” Genes & Development 35: 82–101. 10.1101/GAD.344234.120. 33303642 PMC7778263

[acel70620-bib-0036] Ruan, L. , C. Zhou , E. Jin , et al. 2017. “Cytosolic Proteostasis Through Importing of Misfolded Proteins Into Mitochondria.” Nature 543: 443–446. 10.1038/nature21695. 28241148 PMC5793917

[acel70620-bib-0037] Shen, X. , R. E. Ellis , K. Sakaki , and R. J. Kaisfman . 2005. “Genetic Interactions due to Constitutive Inducible Gene Regulation Mediated by the Unfolded Protein in *C. elegans* .” PLoS Genetics 1: e37. 10.1371/journal.pgen.0010037. 16184190 PMC1231716

[acel70620-bib-0038] Shpilka, T. , and C. M. Haynes . 2018. “The Mitochondrial UPR: Mechanisms, Physiological Functions and Implications in Ageing.” Nature Reviews. Molecular Cell Biology 19: 109–120. 10.1038/nrm.2017.110. 29165426

[acel70620-bib-0039] Taylor, R. C. , and A. Dillin . 2013. “XBP‐1 Is a Cell‐Nonautonomous Regulator of Stress Resistance and Longevity.” Cell 153: 1435–1447. 10.1016/j.cell.2013.05.042. 23791175 PMC4771415

[acel70620-bib-0040] Wang, F. , Y. Dai , X. Zhu , et al. 2021. “Saturated Very Long Chain Fatty Acid Configures Glycosphingolipid for Lysosome Homeostasis in Long‐Lived *C. elegans* .” Nature Communications 12: 5073. 10.1038/s41467-021-25398-6. PMC8379269 34417467

[acel70620-bib-0041] Wang, X. , and X. J. Chen . 2015. “A Cytosolic Network Suppressing Mitochondria‐Mediated Proteostatic Stress and Cell Death.” Nature 524: 481–484. 10.1038/nature14859. 26192197 PMC4582408

[acel70620-bib-0042] Weicksel, S. E. , A. Mahadav , M. Moyle , et al. 2016. “A Novel Small Molecule That Disrupts a Key Event During the Oocyte‐To‐Embryo Transition in *C. elegans* .” Development 143: 3540–3548. 10.1242/dev.140046. 27510972 PMC5087616

[acel70620-bib-0043] Williams, G. C. 1957. “Pleiotropy, Natural Selection and the Evolution of Senescence.” Evolution (New York, N.Y.) 11: 398–411.

[acel70620-bib-0044] Wu, X. , Z. Shi , M. Cui , M. Han , and G. Ruvkun . 2012. “Repression of Germline RNAi Pathways in Somatic Cells by Retinoblastoma Pathway Chromatin Complexes.” PLoS Genetics 8: e1002542. 10.1371/journal.pgen.1002542. 22412383 PMC3297578

[acel70620-bib-0045] Zhang, K. , X. Shen , J. Wu , et al. 2006. “Endoplasmic Reticulum Stress Activates Cleavage of CREBH to Induce a Systemic Inflammatory Response.” Cell 124: 587–599. 10.1016/j.cell.2005.11.040. 16469704

