# Supplementary material for: Proteostasis Rebalancing by LET‐607 Deficiency Promotes Longevity
Source: Aging Cell. 2026 Jul 9;25(7):e70620. doi: 10.1111/acel.70620 (PMC13347320; doi:10.1111/acel.70620)
Supplement: Supplementary file 1 — Figure S1: Additional characterization of LET‐607 depletion and UPRcyto activation. Figure S2: SAMe metabolism mediates UPRcyto regulation upon LET‐607 deficiency. Figure S3: Identification of lin‐61 and H3K9me‐associated chromatin factors in the regulation of UPRcyto. Figure S4: H3K9me loss enhances UPRcyto activation. Figure S5: H3K9 methylation plays a predominant role in the regulation of UPRcyto. Table S1: Survival data. Repeats 1 were graphed in figures. Table S2: qPCR primer sequences. [file ACEL-25-e70620-s001.docx]

**Supplementary Figures and Tables for**

**Proteostasis Rebalancing by LET-607 Deficiency Promotes longevity**

Haixiang Tong^#^, Wei Li^#^, Pangui Yuan^#^, Feng Li, Qin Liu, Shanshan Pang, and Haiqing Tang*


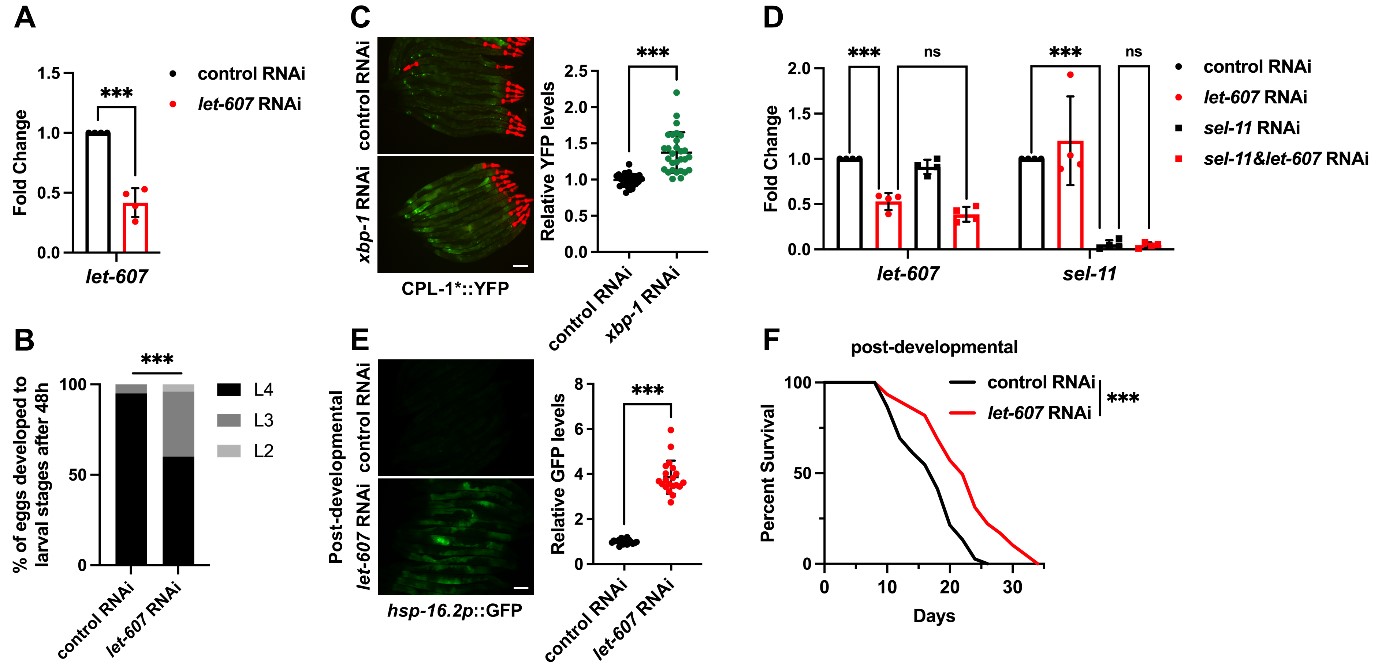


**Figure S1. Additional characterization of LET-607 depletion and UPR^cyto^ activation.**

1. qRT-PCR analysis of *let-607* mRNA levels following *let-607* RNAi treatment. n = 4 independent experiments.
2. Effect of diluted *let-607* RNAi (control RNAi : *let-607* RNAi = 10 : 1) on development. The percentage of animals reaching each larval stage was scored 48 hours after hatching.
3. Effect *xbp-1* RNAi on the expression of CPL-1^W32A;Y35A^::YFP in day 1 adult worms. n = 29 animals. (D) qRT-PCR analysis of *let-607* and *sel-11* mRNA levels following *sel-11* and *let-607* double RNAi treatment. n = 4 independent experiments.
4. Effect of post-developmental *let-607* RNAi on *hsp-16.2p::gfp* expression in day 1 adult worms. n = 20 animals.
5. Effect of post-developmental *let-607* RNAi on lifespan.

Data are presented as mean ± SD.***p < 0.001. Scale bars = 200 µm. (A, C, E) were analyzed by unpaired two-tailed *t* test. (B) was analyzed by Chi-square and Fisher’s exact test. (D) was analyzed by two-way ANOVA with Tukey’s multiple comparisons test. (F) was analyzed by log-rank (Mantel–Cox) test, the additional repeat assays and the corresponding statistical analyses are provided in Table S1.


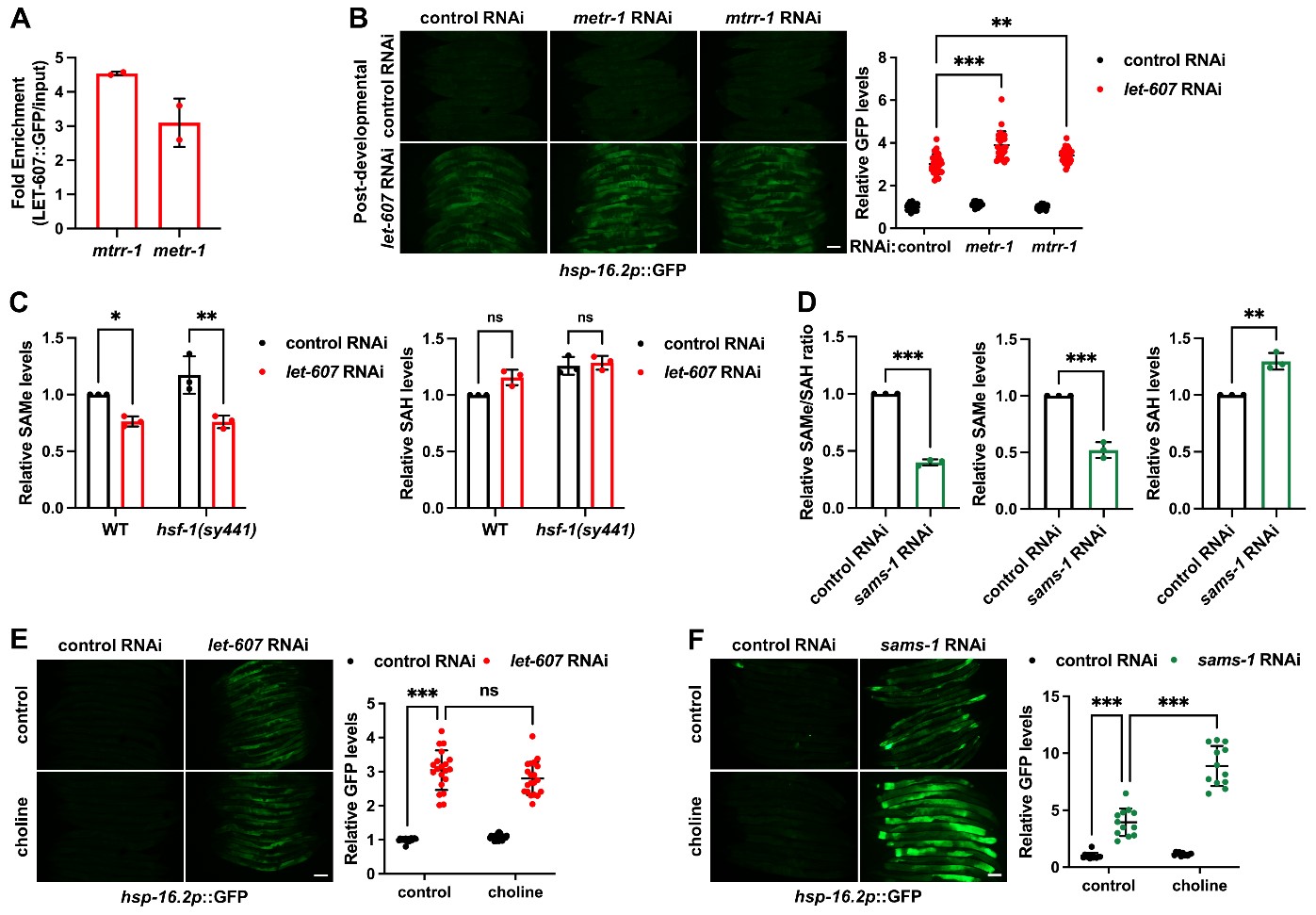


**Figure S2. SAMe metabolism mediates UPR^cyto^ regulation upon LET-607 deficiency.**

1. Fold enrichment of LET-607 ChIP-seq signals at the promoter regions of *mtrr-1* and *metr-1* relative to input controls, based on re-analysis of published datasets (GEO: GSE84419).
2. Effects of post-developmental *let-607* and 1CC gene RNAi on *hsp-16.2p::gfp* expression in day 1 adult worms. n = 20 animals.
3. Effect of *let-607* RNAi on SAMe and SAH levels in WT and *hsf-1(sy441)* mutant worms at day 1

adult stage. n = 3 independent experiments.

1. Effect of *sams-1* RNAi on the SAMe/SAH ratio, SAMe levels, and SAH levels at day 1 adult stage. n = 3 independent experiments.

(E-F) *hsp-16.2p::gfp* expression in *let-607(RNAi)* (E) and *sams-1(RNAi)* (F) worms with or without choline supplementation. n = 20 animals.

Data are presented as mean ± SD. **p* < 0.05, ***p* < 0.01, ***p < 0.001. Scale bars = 200 µm. (B, C, E, F) were analyzed by two-way ANOVA with Tukey’s multiple comparisons test. (D) was analyzed by unpaired two-tailed *t* test.


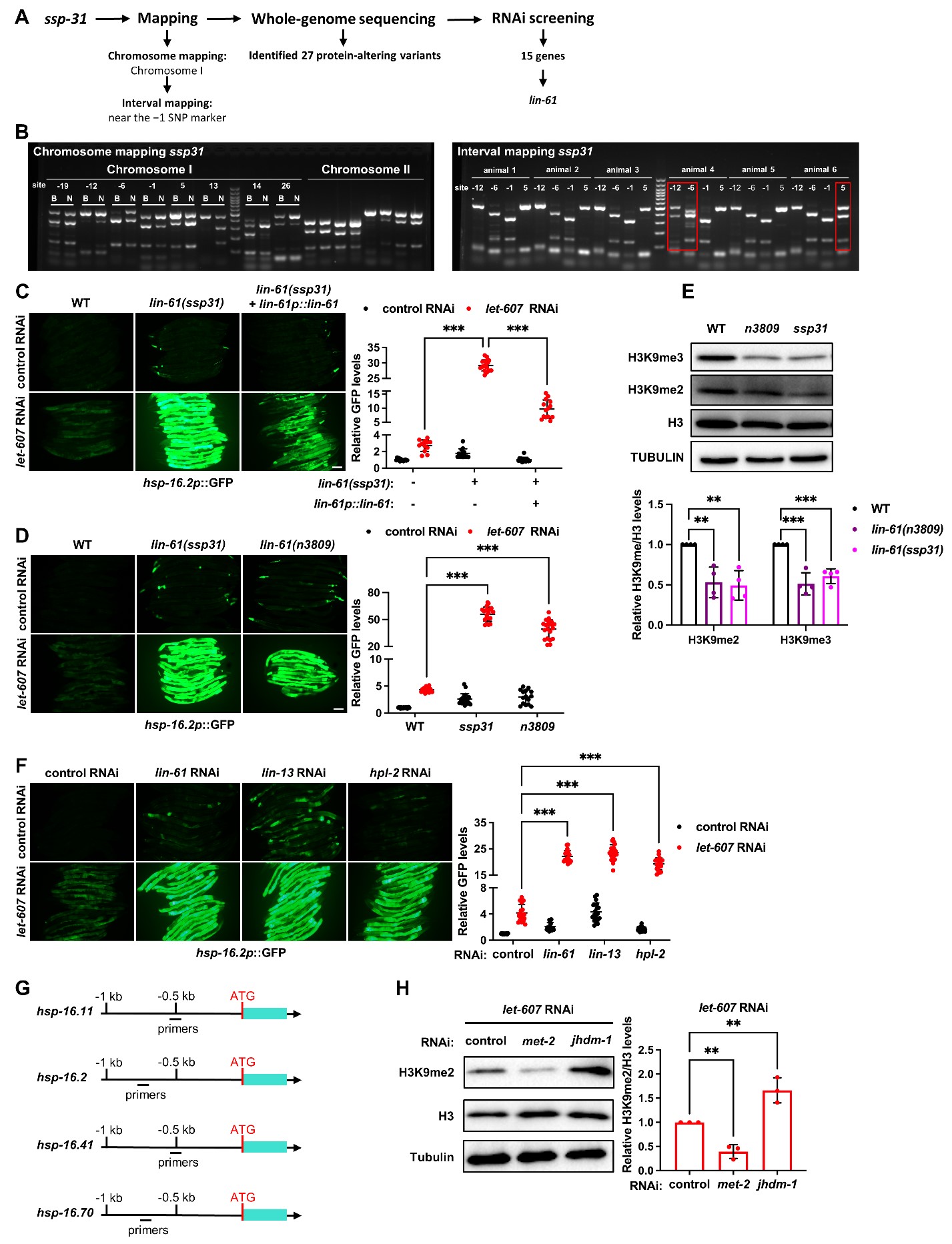


**Figure S3. Identification of *lin-61* and H3K9me-associated chromatin factors in the regulation of**

**UPR^cyto^.**

1. Schematic workflow for identification of the *ssp31* mutation.
2. SNP mapping of *ssp31* mutation. Chromosome mapping: Each pair of lanes shows results from the SNP at the indicated genetic map position, using either the Brighter GFP (B) or the Normal GFP (N) template. Distinct SNP segregation patterns between the Brighter GFP and Normal GFP pools were observed specifically on chromosome I between the −12 and 5 SNP markers, whereas SNP patterns on other chromosomes were comparable between the two groups, indicating linkage of *ssp31* to this interval. Interval mapping: Each column represents an individual Brighter GFP recombinant assayed for four SNP markers (−12, −6, −1, and 5). All recombinants retained Bristol N2 DNA at the −1 SNP marker, indicating tight linkage to this region, whereas Hawaiian DNA was detected at the −12, −6, and 5 markers in multiple recombinants (highlighted by red boxes). These results further localized *ssp31* to the vicinity of the −1 SNP marker.
3. Effect of *lin-61* expression vector on *hsp-16.2p::gfp* expression in *lin-61(ssp31);let-607(RNAi)* mutants. n = 13-17 animals.
4. Effects of *lin-61(ssp31)* and *lin-61(n3809)* mutations on *hsp-16.2p::gfp* expression during *let-607* RNAi at day 1 adulthood. n = 20 animals.
5. Western blot analysis of H3K9me2 and H3K9me3 levels in *lin-61(ssp31)* and *lin-61(n3809)* day 1 adult worms. n = 4 independent experiments.
6. Effects of RNAi targeting *lin-61*, *lin-13*, and *hpl-2* on *hsp-16.2p::gfp* expression in day 1 adult animals under *let-607* RNAi. n = 20 animals.
7. Location of the ChIP–qPCR primers for UPR^cyto^ genes.
8. Effects of *met-2* and *jhdm-1* RNAi on H3K9me2 levels in *let-607(RNAi)* animals at day 1 adulthood. n = 3 independent experiments.

Data are presented as mean ± SD. ***p* < 0.01, ****p* < 0.001. Scale bars = 200 µm. (C, D, F) were analyzed by two-way ANOVA with Tukey’s multiple comparisons test. (E) was analyzed by multiple *t* tests with Holm–Šidák correction for multiple comparisons. (H) was analyzed by one-way ANOVA with

Dunnett’s multiple comparisons test.


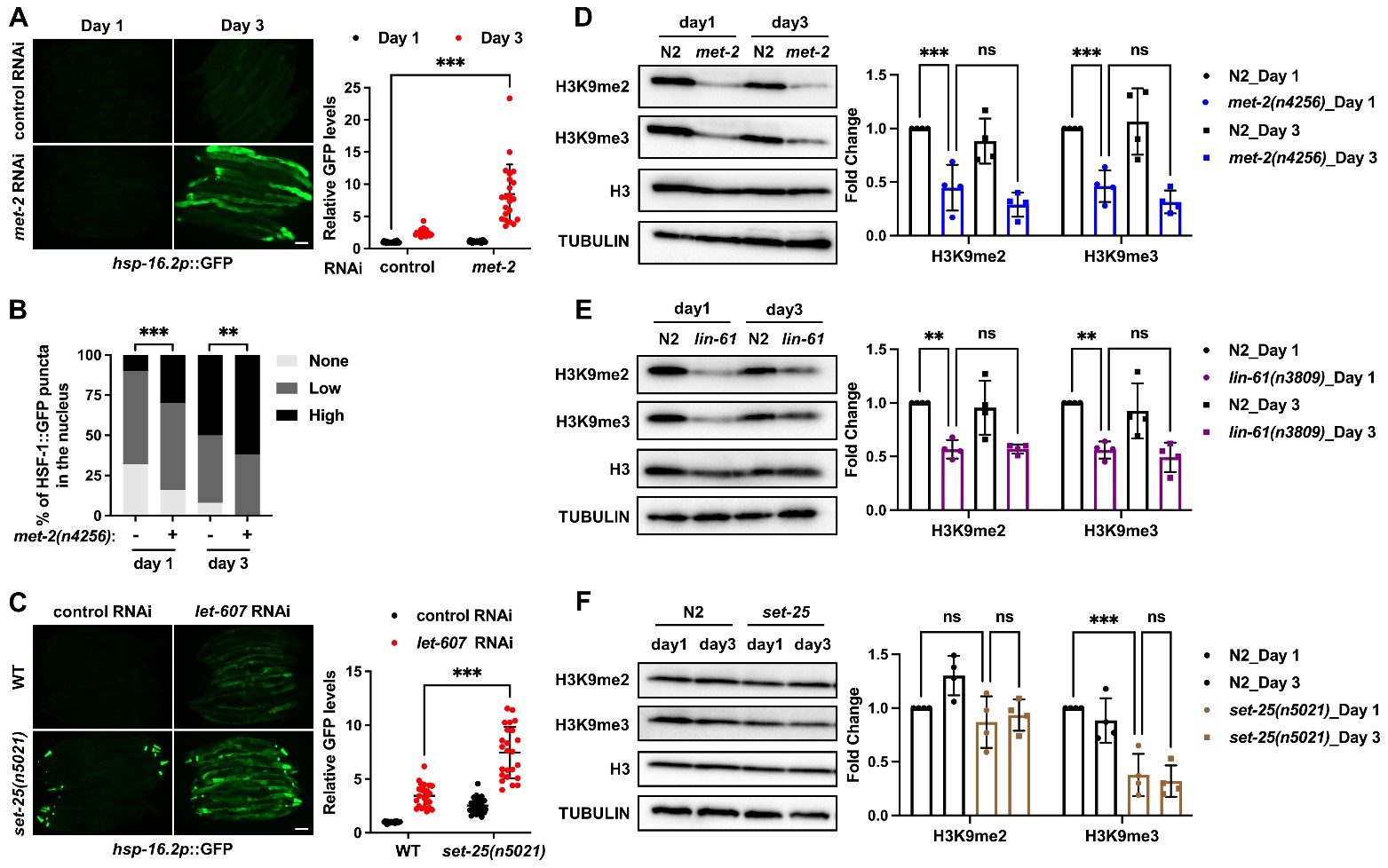


**Figure S4. H3K9me loss enhances UPR^cyto^ activation**

(A) Effect of *met-2* RNAi on *hsp-16.2p::gfp* expression in day 1 and day 3 adults. n = 22 animals. (B) Effect of *met-2* mutation on HSF-1::GFP nuclear puncta in day 1 and day 3 adults. n = 15-25 animals.

(C) Effect of *set-25* mutation on *hsp-16.2p::gfp* expression during *let-607* RNAi at day 1 adulthood. n = 24 animals.

(D–F) Western blot analysis of H3K9me2 and H3K9me3 levels in WT, *met-2(n4256)* (D), *lin-61(n3809)* (E), and *set-25(n5021)* (F) mutants at day 1 and day 3 adulthood. n = 4 independent experiments. Data are presented as mean ± SD. ***p* < 0.01, ****p* < 0.001. Scale bars = 200 µm. (A, C, D, E, F) were analyzed by two-way ANOVA with Tukey’s multiple comparisons test. (B) was analyzed by Chi-square and Fisher’s exact test.


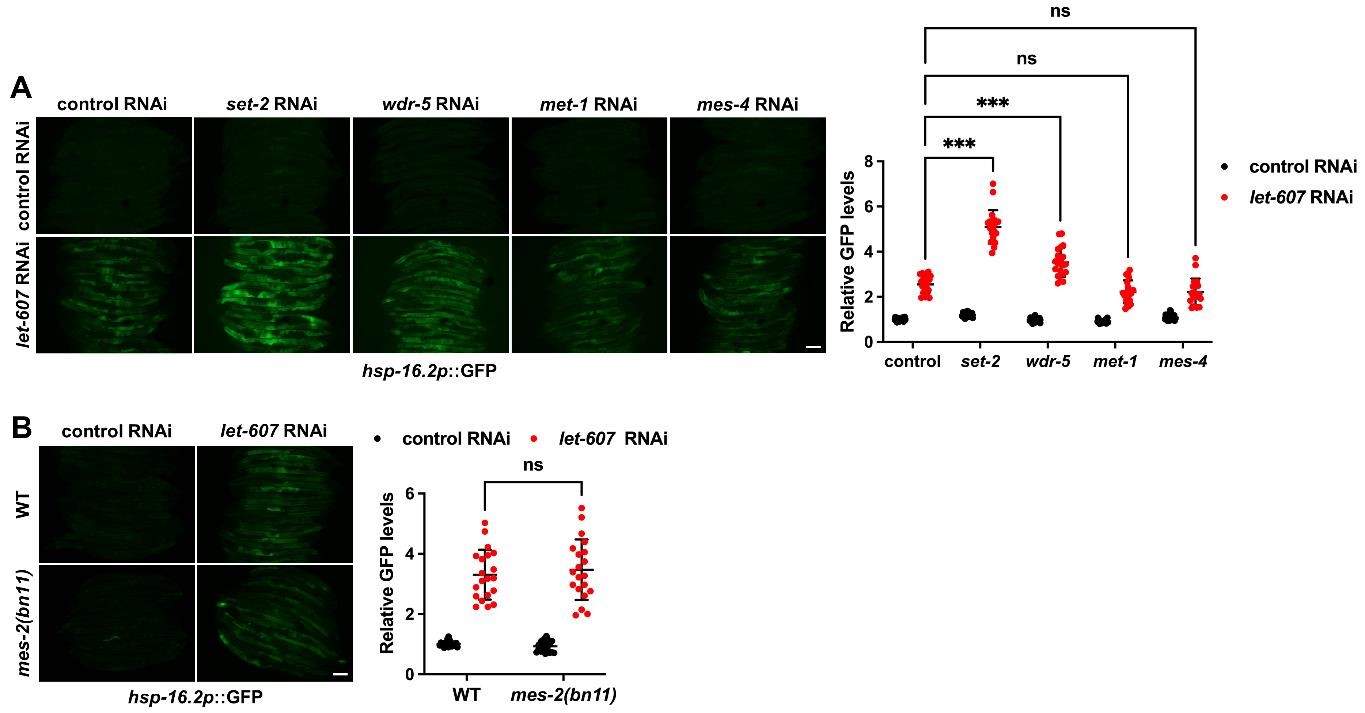


**Figure S5. H3K9 methylation plays a predominant role in the regulation of UPR^cyto^.**

1. Effects of RNAi targeting H3K4 methyltransferases (*set-2* and *wdr-5*) and H3K36

methyltransferases (*met-1* and *mes-4*) on *hsp-16.2p::gfp* expression during *let-607* RNAi in day 1 adult worms. n = 20 animals.

1. Effect of the H3K27 methyltransferase *mes-2* mutation on *hsp-16.2p::gfp* expression during *let-607* RNAi in day 1 adult worms. n = 20 animals.

Data are presented as mean ± SD. ****p* < 0.001. Scale bars = 200 µm. (A, B) were analyzed by two-way ANOVA with Tukey’s multiple comparisons test.

**Table S1. Survival data. Repeats 1 were graphed in figures.**

| **Figures** | **Strain/Treatment** | **Mean survival time ± SEM (hours/days)** | **# Worms Censored/Total** | **Bonferroni P value** |
| --- | --- | --- | --- | --- |
| 1J |  |  |  |  |
| **Repeat 1** | **WT control RNAi** | **12.29±0.43** | **6/107** |  |
|  | **WT *let-607* RNAi** | **22.74±0.69** | **4/109** | ＜**0.0001^a^** |
|  | ***sy441* control RNAi** | **14.16±0.44** | **7/99** |  |
|  | ***sy441 let-607* RNAi** | **15.56±0.36** | **6/105** | **0.2792 ^a^** |
| **Repeat 2** | **WT control RNAi** | **15.6±0.51** | **0/114** |  |
|  | **WT *let-607* RNAi** | **21.84±0.42** | **5/111** | ＜**0.0001^a^** |
|  | ***sy441* control RNAi** | **15.42±0.27** | **3/117** |  |
|  | ***sy441 let-607* RNAi** | **15.3±0.28** | **9/120** | **1a** |
| **Repeat 3** | **WT control RNAi** | **13.88±0.37** | **6/119** |  |
|  | **WT *let-607* RNAi** | **22.3±0.55** | **3/129** | ＜**0.0001^a^** |
|  | ***sy441* control RNAi** | **14.8±0.36** | **6/115** |  |
|  | ***sy441 let-607* RNAi** | **15.96±0.44** | **6/114** | **0.016^a^** |
| S1F |  |  |  |  |
| **Repeat 1** | **WT control RNAi** | **17.03±0.55** | **6/82** |  |
|  | **WT *let-607* RNAi** | **22.16±0.7** | **5/81** | ＜**0.0001^a^** |
| **Repeat 2** | **WT control RNAi** | **17.64±0.5** | **6/84** |  |
|  | **WT *let-607* RNAi** | **20.77±0.67** | **5/83** | ＜**0.0001^a^** |
| **Repeat 3** | **WT control RNAi** | **18.05±0.47** | **3/84** |  |
|  | **WT *let-607* RNAi** | **21.77±0.65** | **3/81** | ＜**0.0001^a^** |
| 5A |  |  |  |  |
| **Repeat 1** | **WT** | **14.52 ± 0.32** | **5/118** |  |
|  | ***lin-61(n3809)*** | **18.17 ± 0.48** | **3/110** | ＜**0.0001^a^** |

| **Repeat 2** | **WT** | | **14.59 ± 0.2** | **10/206** | |  |
| --- | --- | --- | --- | --- | --- | --- |
|  | ***lin-61(n3809)*** | | **16.77 ± 0.39** | **20/176** | | ＜**0.0001^a^** |
| **Repeat 3** | **WT** | | **14.93 ± 0.29** | **8/101** | |  |
|  | ***lin-61(n3809)*** | | **18.14 ± 0.37** | **20/182** | | ＜**0.0001^a^** |
| 5B |  | |  |  | |  |
| **Repeat 1** | **control RNAi** | | **15.94 ± 0.4** | **11/124** | |  |
|  | ***lin-61* RNAi** | | **18.36 ± 0.53** | **10/109** | | **0.0001^a^** |
|  | ***met-2* RNAi** | | **19.78 ± 0.42** | **4/147** | | ＜**0.0001^a^** |
|  | ***set-25* RNAi** | | **20.79 ± 0.38** | **11/155** | | ＜**0.0001^a^** |
| **Repeat 2** | **control RNAi** | | **16.64 ± 0.42** | **11/130** | |  |
|  | ***lin-61* RNAi** | | **18.92 ± 0.63** | **12/100** | | **0.0017^a^** |
|  | ***met-2* RNAi** | | **19.03 ± 0.55** | **13/140** | | **0.0002^a^** |
|  | ***set-25* RNAi** | | **19.63 ± 0.57** | **15/91** | | **0.0002^a^** |
| **Repeat 3** | **control RNAi** | | **17.83 ± 0.47** | **8/114** | |  |
|  | ***lin-61* RNAi** | | **20.57 ± 0.52** | **20/125** | | ＜**0.0001^a^** |
|  | ***met-2* RNAi** | | **21 ± 0.46** | **13/104** | | ＜**0.0001^a^** |
|  | ***set-25* RNAi** | | **21.13 ± 0.41** | **18/114** | | ＜**0.0001^a^** |
| 5C |  | |  |  | |  |
| **Repeat 1** | **WT** | | **8.66 ± 0.14** | **0/61** | |  |
|  | ***lin-61(n3809)*** | | **10.48 ± 0.14** | **0/100** | | ＜**0.0001^a^** |
| **Repeat 2** | **WT** | | **8.89 ± 0.21** | **3/85** | |  |
|  | ***lin-61(n3809)*** | | **10.21 ± 0.18** | **0/78** | | ＜**0.0001^a^** |
| **Repeat 3** | **WT** | | **8.61 ± 0.15** | **0/66** | |  |
|  | ***lin-61(n3809)*** | | **9.79 ± 0.15** | **1/78** | | ＜**0.0001^a^** |
| 5D |  | |  |  | |  |
| **Repeat 1** | **WT control RNAi** | | **17.07±0.33** | **1/151** | |  |
|  | **WT *hsf-1* RNAi** | | **14.41±0.23** | **0/137** | |  |
|  | ***lin-61(n3809)*** | | **19.61±0.43** | **2/127** | | ＜**0.0001^a^** |
|  | ***lin-61 hsf-1* RNAi** | **11.66±0.13** | | | **0/169** | ＜**0.0001^a^** |
| **Repeat 2** | **WT control RNAi** | **17.33±0.22** | | | **2/205** |  |
|  | **WT *hsf-1* RNAi** | **14.31±0.3** | | | **0/261** |  |
|  | ***lin-61(n3809)*** | **20.4±0.22** | | | **3/227** | ＜**0.0001^a^** |
|  | ***lin-61 hsf-1* RNAi** | **13.11±0.19** | | | **0/326** | ＜**0.0001^a^** |
| **Repeat 3** | **WT control RNAi** | **15.99±0.25** | | | **2/273** |  |
|  | **WT *hsf-1* RNAi** | **12.36±0.29** | | | **0/155** |  |
|  | ***lin-61(n3809)*** | **18.68±0.25** | | | **5/267** | ＜**0.0001^a^** |
|  | ***lin-61 hsf-1* RNAi** | **10.61±0.18** | | | **1/230** | ＜**0.0001^a^** |

a vs same strain

**Table S2. qPCR primer sequences.**

| Primers | Sequences |
| --- | --- |
| *snb-1* Forward | GCAAGTATTGGTGGAAGA |
| *snb-1* Reverse | ACGATGATGATAATAAGAATGAC |
| *hsp-16.11* Forward | GCAGAGGCTCTCCATCTGAA |
| *hsp-16.11* Reverse | GCTTGAACTGCGAGACATTG |
| *hsp-16.2* Forward | ACCTGAAGATGTAGATGTTG |
| *hsp-16.2* Reverse | TTGCCTGTTGAATTGGAA |
| *hsp-16.41* Forward | ATCGGAACATGGATACTTGA |
| *hsp-16.41* Reverse | AATGGCAGATTTGACAGAAG |
| *hsp-70* Forward | CCGTTGTTGAGGTTGAAG |
| *hsp-70* Reverse | CACAGTAATGACAGCATCC |
| *ire-1* Forward | TGGAAACTCTATCATCAGCGT |
| *ire-1* Reverse | CCACGTATTCACTTCAGGC |
| *mel-32* Forward | CACCAACAACGAGAACATT |
| *mel-32* Reverse | GCATCCATAACAGCCTTG |
| *Y56A3A.19* Forward | TCCAACAACGAGTCATCA |

| *Y56A3A.19* Reverse | CAACACAATTCGCTCCTC |
| --- | --- |
| *xbp-1s* Forward | TGCCTTTGAATCAGCAGTGG |
| *xbp-1s* Reverse | ACCGTCTGCTCCTTCCTCAATG |
| *rpn-11* Forward | CGTCACTACTACTCCATCC |
| *rpn-11* Reverse | TGCTCCTTGTTCTGTTCA |
| *lev-11* Forward | CCAGAAGAAGATGACTCAGA |
| *lev-11* Reverse | GTTCAAAGAAGCGACCTC |
| *pek-1* Forward | ATGGAGGATCTGACAGAACT |
| *pek-1* Reverse | CTCAATTCCTCCTGATGAAGAG |
| *eif-2α* Forward | CGTATTTACTAGGGTGGTCGTC |
| *eif-2α* Reverse | GCCTATGCTTATTTGTGTTCGG |
| *atf-4* Forward | CCATTCCACCCCACAATAT |
| *atf-4* Reverse | GGAAGTTGACATCGGAGTT |
| *atf-6* Forward | GAATCACAAGAATCGACCTCT |
| *atf-6* Reverse | CCCACATTTCCTGGTCAT |
| *acdh-1* Forward | CGTCTCTGTTCTGATAGTCT |
| *acdh-1* Reverse | TGCCTCTCCTGAATTAGTAAT |
| *flcn-1* Forward | AGTTGGCGAATCTCAAGA |
| *flcn-1* Reverse | CCAGTGACAATATGACGAATT |
| *gcsh-1* Forward | GTTGGCTTTACAGACTTGAA |
| *gcsh-1*  Reverse | AACTTGTTATATTGCTCCTCAG |
| *mthf-1* Forward | TCATCAACTACATTACTCAAGC |
| *mthf-1*  Reverse | AATTCCATTCTCATTACACCAA |
| *mtrr-1* Forward | ATGAAGGAAGAGGAAGAGTTAT |
| *mtrr-1*  Reverse | ACGAGACGATGAAAGGTT |
| *metr-1* Forward | TTACGATACGGCGATTGA |
| *metr-1*  Reverse | GAGGATATGAGACGAAGGAA |
| *sams-1* Forward | CAAGGACGGAGAAGATGT |
| *sams-1*  Reverse | TGTGGGAAAGAATGAGAGT |
| ChIP *hsp-16.11* Forward | TGAGAAACATCGAGTTGAACAGAGA |
| ChIP *hsp-16.11* Reverse | GCTCATGCTCCGTTCTCCAT |
| ChIP *hsp-16.2* Forward | GAAATGGCAGATTTGACAGAAGGT |
| ChIP *hsp-16.2* Reverse | ATTGGGGAGGTAAGTAAGCATCA |
| ChIP *hsp-16.41* Forward | TCTCTGAGCTGTTTGACTGGG |
| ChIP *hsp-16.41* Reverse | CAGTCGCTTTACGGACGATGA |
| ChIP *hsp-70* Forward | AATGTTCCCCAAACGCAGATTT |
| ChIP *hsp-70* Reverse | TCATCGAACGTTTGCAAGAGAAG |
